# Factors influencing the acceptability of remote multicomponent rehabilitation versus standard care following critical illness: a theory-informed qualitative study embedded within the iRehab trial

**DOI:** 10.1186/s40560-026-00908-0

**Published:** 2026-07-26

**Authors:** Cara G. Ghiglieri, Bronwen A. Connolly, Darren Murphy, Daniel F. McAuley, Brenda M. O’Neill, Judy M. Bradley

**Affiliations:** 1 Wellcome-Wolfson Institute for Experimental Medicine, School of Medicine, Dentistry and Biomedical Science, Queen’s University Belfast, Belfast, Northern Ireland, UK; 2 Institute of Nursing and Health Research, Ulster University, Belfast, Northern Ireland, UK; 3 Department of Physiotherapy, The University of Melbourne, Melbourne, Australia

**Keywords:** Critical illness, Rehabilitation, Telemedicine, Patient acceptance of health care, Qualitative research

## Abstract

**Background:**

Adults who survive critical illness requiring mechanical ventilation frequently experience prolonged physical, psychological, and social difficulties following discharge, yet access to structured recovery support remains variable. This study explored how ICU survivors and staff perceived the acceptability of a remote multicomponent rehabilitation programme versus standard care following discharge from critical care.

**Methods:**

A theory-informed qualitative interview study was conducted, guided by the Theoretical Framework of Acceptability. ICU survivors who received either remote multicomponent rehabilitation or standard care, alongside staff involved in delivery, were purposively sampled from a pragmatic UK trial (iRehab) across 24 hospital sites. Semi-structured interviews were audio-recorded, transcribed verbatim, and analysed using framework analysis. Thirty-five ICU survivors and six staff members participated in interviews conducted between January 2023 and June 2025.

**Results:**

Eight inter-related themes captured the factors shaping acceptability during recovery after ICU discharge. Participants who received remote multicomponent rehabilitation generally found it acceptable, as it provided structure, continuity, and support for confidence-building and self-management. In contrast, standard care was often experienced as lacking coordination and follow-up, leaving individuals to navigate recovery with limited support. However, acceptability was not experienced in the same way by all participants. Digital delivery and optional components, such as peer support, were valued by many but not by everyone, depending on readiness, context, and capability.

**Conclusions:**

Remote multicomponent rehabilitation was valued for its structure, continuity, and support, while standard care was often experienced as lacking these features. Acceptability of support was shaped by how well it aligned with survivors’ needs and contexts. These findings support the integration of structured, flexible rehabilitation pathways into post-ICU care.

*Trial registration* ISRCTN11266403; registered 26 July 2022.

**Supplementary Information:**

The online version contains supplementary material available at 10.1186/s40560-026-00908-0.

## Background

Of the thousands of individuals discharged from hospitals in the United Kingdom each year following an episode of critical illness [1], many experience persistent physical, cognitive, and psychological difficulties such as muscle weakness, fatigue, anxiety, and post-traumatic stress [2–4]. These complications can be prolonged, limiting independence [5], reducing health-related quality of life (HRQoL) [6] and increasing healthcare utilisation [7]. Approximately one in four survivors are readmitted within 90 days of discharge [8]. The broader societal impact is considerable, with nearly half of survivors unable to return to work within a year and many requiring ongoing community or social care support [9, 10].

Rehabilitation is a cornerstone of recovery following critical illness; however, access to structured post-ICU rehabilitation remains inconsistent across the United Kingdom [11]. While some survivors benefit from coordinated, multidisciplinary rehabilitation, others are discharged with minimal or no structured rehabilitation or follow-up, resulting in significantly heterogeneous recovery experiences [11]. In response, remote and hybrid models of structured rehabilitation are gaining prominence [12–14], reflecting the evolving diversity in how post**-**ICU recovery is organised, delivered, and experienced.

Recovery from critical illness is a process shaped by personal, social, and systemic factors [6, 15, 16]. Survivors often require ongoing guidance and feedback, yet many encounter fragmented or minimal support, leaving them to manage recovery largely independently [17]. To date, most research has focused on identifying factors that facilitate or hinder recovery [18] with less attention paid to how survivors perceive the acceptability of the support available to them [19]. Acceptability refers to the extent to which an intervention is considered appropriate based on individuals’ cognitive and emotional responses [19]. Understanding both recipient and provider perspectives is crucial, as these appraisals influence engagement, adherence, and ultimately perceived effectiveness. The Theoretical Framework of Acceptability (TFA) provides a structured approach to examining these responses across multiple domains, including affective attitude, burden, ethicality, intervention coherence, opportunity costs, perceived effectiveness, and self-efficacy [19].

For this reason, acceptability was assessed as a secondary outcome in the iRehab trial, a multicentre RCT evaluating a 6-week, remotely delivered multicomponent personalised rehabilitation intervention for people discharged home following an Intensive Care Unit (ICU) admission, compared with standard care, where structured rehabilitation is not routinely provided [20]. Findings indicated that participants receiving remote rehabilitation viewed the support as more acceptable than those receiving standard care. While these findings provide a quantitative indication of relative acceptability, qualitative exploration offers further insight into how acceptability was experienced in practice and which features of rehabilitation and standard care shaped participants’ appraisals over time. This study reports these qualitative findings.

## Methods

### Aim

The aim of this study was to explore how ICU survivors and staff perceived the acceptability of a remote multicomponent rehabilitation programme versus standard care following discharge from critical care.

### Study design

This study was a theoretically informed qualitative study, guided by the Theoretical Framework of Acceptability (TFA), and embedded within the iRehab trial [21]. The iRehab trial was prospectively registered prior to participant enrollment with ISRCTN (ISRCTN11266403; registered 26 July 2022). The study adhered to the Consolidated Criteria for Reporting Qualitative Research (COREQ) guidelines (Additional file 1).

### Intervention and standard care (iRehab)

The iRehab intervention comprised a 6-week multicomponent programme delivered remotely by iRehab specialists. It included weekly one-to-one appointments focused on symptom management, psychological support, goal setting and review, and individually tailored exercise and activity planning. These components were adapted and progressed according to individual ability and goals. Participants were also offered independent, recorded or live group exercise sessions and access to the iRehab Peer Support Café. One-to-one appointments were delivered via Microsoft Teams or telephone according to participant preference and access.

Standard care comprised usual NHS care available at the recruiting site, with no additional structured rehabilitation provided by the trial. Sites offering structured post-ICU rehabilitation comparable to iRehab were not eligible to participate. Standard care typically involved variable follow-up arrangements (typically a single brief contact), ad hoc support, and referral or signposting to existing services, rather than a defined multicomponent rehabilitation programme. 

### Study participants

Eligibility was limited to iRehab trial participants, briefly, adult ICU survivors who had received invasive mechanical ventilation for at least 48 h and been discharged from hospital within the previous 12 weeks. Participants also needed to be able to engage in the intervention (e.g., via computer or telephone) and understand spoken English [21].

Purposive sampling included both trial arms (remote rehabilitation versus standard care) and sought variation in gender, age, region, hospital site, and mode of engagement. A target of 35–40 participants was set, consistent with recommendations for multisite Framework Method studies [22]. Sampling and analysis proceeded iteratively. Following completion of 35 patient interviews, the research team undertook a planned review of the dataset to assess informational depth [23] and cross-group variation. At this point, the dataset was judged sufficiently rich to address the research question, and recruitment, therefore, concluded at 35 patient interviews, within the target sample. All iRehab delivery staff were also invited to contribute to capture professional perspectives on the acceptability of delivering the intervention.

### Data collection

Patients were invited to interview after completing the 8-week outcome assessment appointment within the iRehab trial. Eligible and willing participants received an information sheet by email and were then contacted by telephone after 48 h to confirm interest. Written or verbal consent was obtained before interview.

Semi**-**structured interviews were conducted by two female postdoctoral researchers between January 2023 and June 2025 via Microsoft Teams or telephone, according to participant preference. Interviews averaged 58 min (range 31**–**84). No prior relationship existed between interviewer and participant. While a few participants had relatives present, only participants were interviewed and all responses were their own. Participants were aware that the researcher was part of the wider study and sought to understand recovery experiences following critical illness.

Interview guides (Additional files 2–3) were theory-informed, drawing on the Theoretical Framework of Acceptability [19], to explore perceptions of remote rehabilitation and standard care. Before the interview, participants completed the Theoretical Framework of Acceptability Questionnaire (TFAQ) [24] that was modified for iRehab context. Interviews were audio-recorded and transcribed verbatim by a Queen’s University Belfast-approved transcription service.

### Data analysis

Qualitative analysis was conceptually guided by the TFA, while TFAQ data were analysed descriptively to contextualise qualitative findings.

### TFAQ analysis

Participants completed a modified TFAQ [24] using a five-point Likert scale tailored to each of the seven TFAQ domains (additional files 4–6). Scores ranged from 1 (low) to 5 (high) acceptability, with burden and opportunity cost domains reverse scored. Mean and standard deviation scores were calculated for each TFAQ domain to maintain consistency with the parent iRehab trial, in which TFAQ scores were analysed as continuous secondary outcomes. An overall acceptability score was derived by averaging these. A separate single-item global rating summarised general perceptions of acceptability. These descriptive data complement qualitative interview findings by providing contextual insight.

### Interview analysis

Interview data, including audio recordings and transcripts, were analysed using a combined inductive–deductive approach to the Framework Method [22]. The method was chosen for its structured yet flexible design, enabling in-depth comparison across individual experiences and professional perspectives [22, 25]. Following familiarisation, two researchers independently conducted line-by-line coding of eight transcripts selected to represent remote rehabilitation, standard care and staff accounts. Double coding was used to support framework development and interpretive consistency rather than to quantify coder agreement. Coding discussions focused primarily on interpretation, code boundaries and organisation of the analytical framework, rather than disagreement regarding whether concepts were present in the data.

Codes were compared, refined, and organised into separate working analytical frameworks for each dataset, which were then compared and merged into a final analytical framework covering all groups. Distinctions were retained, where merging risked losing important differences in meaning. The final framework was organised and interpreted in relation to the TFA, while codes that did not align with the TFA were retained to capture contextual and emergent aspects of recovery.

The final framework was applied across all transcripts and charted into a matrix to support within- and cross-case comparison. Themes were developed iteratively through team discussion. NVivo supported data management. Quotations are labeled using anonymised codes, for example, RR11-remote rehabilitation and SC04-standard care, and presented verbatim, with ellipses […] indicating minor omissions for readability. Full analytic detail is provided in Additional file 7.

### Rigour

Several procedures enhanced study rigour and trustworthiness. The TFA informed interview guide development to ensure conceptual consistency, while the combined inductive and deductive approach enabled unanticipated insights to be retained alongside theory-informed interpretation. Researchers with extensive qualitative experience conducted semi-structured interviews to promote depth and comparability. Collaborative coding, regular team discussions, and an audit trail supported reflexivity, transparency, and consistency of interpretation. Illustrative quotations enhanced credibility, and findings were situated within existing literature to support transferability.

## Results

In total, 90 participants were approached during their 8-week outcome assessment appointment; 26 declined participation, and 64 consented to be contacted. Of these, 35 patient interviews were conducted. Not all participants who agreed to be contacted ultimately participated, reflecting practical reasons such as unsuccessful attempts to establish contact or being outside the planned post-trial interview period at the time of contact.

All 20 participants included in this qualitative sub-study from the remote rehabilitation group had completed the core intervention as planned. Completion was defined a priori as attendance at five or more one-to-one appointments with an iRehab specialist. Use of the optional group-based exercise sessions and Peer Support Café varied across participants; 10/20 participants (50%) attended the Exercise Class and/or Café on at least one occasion. Attendance at these optional components was treated as component engagement, rather than a requirement for completion of the full programme. All staff involved in delivering the intervention were invited; six of seven participated, with one completing an additional follow-up interview. Participant demographic and clinical characteristics are presented in Tables 1 and 2.

**Table 1:** Sociodemographic and clinical characteristics of patient participants

Characteristic	Remote Rehabilitation (*n* = 20)	Standard care (*n* = 15)
Gender		
Female	7	6
Male	13	9
Age, years (mean ± SD; range)	56.2 ± 17.0 (23–77)	66.1 ± 9.3 (41–77)
Ethnicity		
White	17	15
Pakistani	1	0
Chinese	1	0
Greek Cypriot	1	0
Time post-ICU discharge, days (mean ± SD; range)	138.1 ± 41.8 (79–207)	135.9 ± 33.5 (87–202)
ICU length of stay, days (mean ± SD; range)	19.6 ± 18.4 (2–68)	14.0 ± 10.6 (2–44)
Reason for ICU admission		
Respiratory system	6	6
Surgical/medical procedures	3	3
Sepsis/infection	2	3
Gastrointestinal (GIT)	2	1
Cardiovascular system	2	0
Endocrine	2	0
Orthopaedic/Trauma	1	0
Central nervous system	1	0
Renal and urinary	0	1
Hepatobiliary	0	1
Systemic lupus erythematosus (multisystem)	1	0
Employment status before ICU		
Retired	5	9
Working full-time (≥ 30 h/week)	6	1
Working part-time (< 30 h/week)	3	4
Unable to work (illness/disability)	4	1
Unemployed but looking for work	2	0
Living arrangements		
Living alone	4	4
Living with others	16	11

**Table 2:** Characteristics of staff participants

Characteristic	Staff participants (*n* = 6)
Gender	
Female	5
Male	1
Ethnicity	
White	5
Asian	1

### TFAQ responses

Thirty-three patients completed the TFAQ during or before interview, including 19 participants in the remote rehabilitation arm and 14 in standard care. Two participants did not complete the questionnaire. All staff participants completed the TFAQ. Data are reported in Table 3. Participants receiving remote rehabilitation reported higher acceptability across most domains, particularly affective attitude, coherence, and perceived effectiveness. Staff ratings were also high, although perceived burden was lower among staff.

**Table 3:** TFAQ domain, general, and single acceptability scores for all participants

TFAQ domain	Remote rehabilitation (*n* = 19) Mean (SD)	Standard care (*n* = 14) Mean (SD)	Staff (*n* = 6) Mean (SD)
Affective attitude	4.53 (0.51)	3.50 (1.29)	4.50 (0.58)
Burden (reversed)	3.79 (1.08)	3.71 (1.20)	2.67 (1.03)
Ethicality	4.42 (0.69)	3.50 (1.45)	4.17 (0.75)
Perceived effectiveness	4.47 (0.51)	3.43 (1.28)	4.00 (0.63)
Coherence	4.47 (0.77)	3.00 (1.52)	4.33 (0.51)
Self-efficacy	4.21 (0.85)	3.86 (0.86)	4.00 (0.00)
Opportunity cost (reversed)	4.11 (1.10)	4.07 (0.62)	4.00 (0.63)
General acceptability	4.74 (0.45)	3.64 (1.28)	4.00 (0.00)
Single acceptability*	4.29 (0.40)	3.58 (0.98)	3.95 (0.14)

*Single acceptability = mean of the seven domains, excluding ‘General acceptability’

### Interview findings

Eight themes were identified across patient and staff accounts of recovery following critical illness, spanning both structured remote rehabilitation and standard care. These themes reflect how participants appraised the acceptability of these forms of support within the broader context of post-ICU recovery.

The first theme, *Early recovery—falling through gaps in follow-up*, was developed inductively and captures the contextual conditions shaping subsequent appraisals of acceptability. The remaining seven themes reflect participants’ evaluations mapped to the constructs of the TFA.

**Fig. 1:**
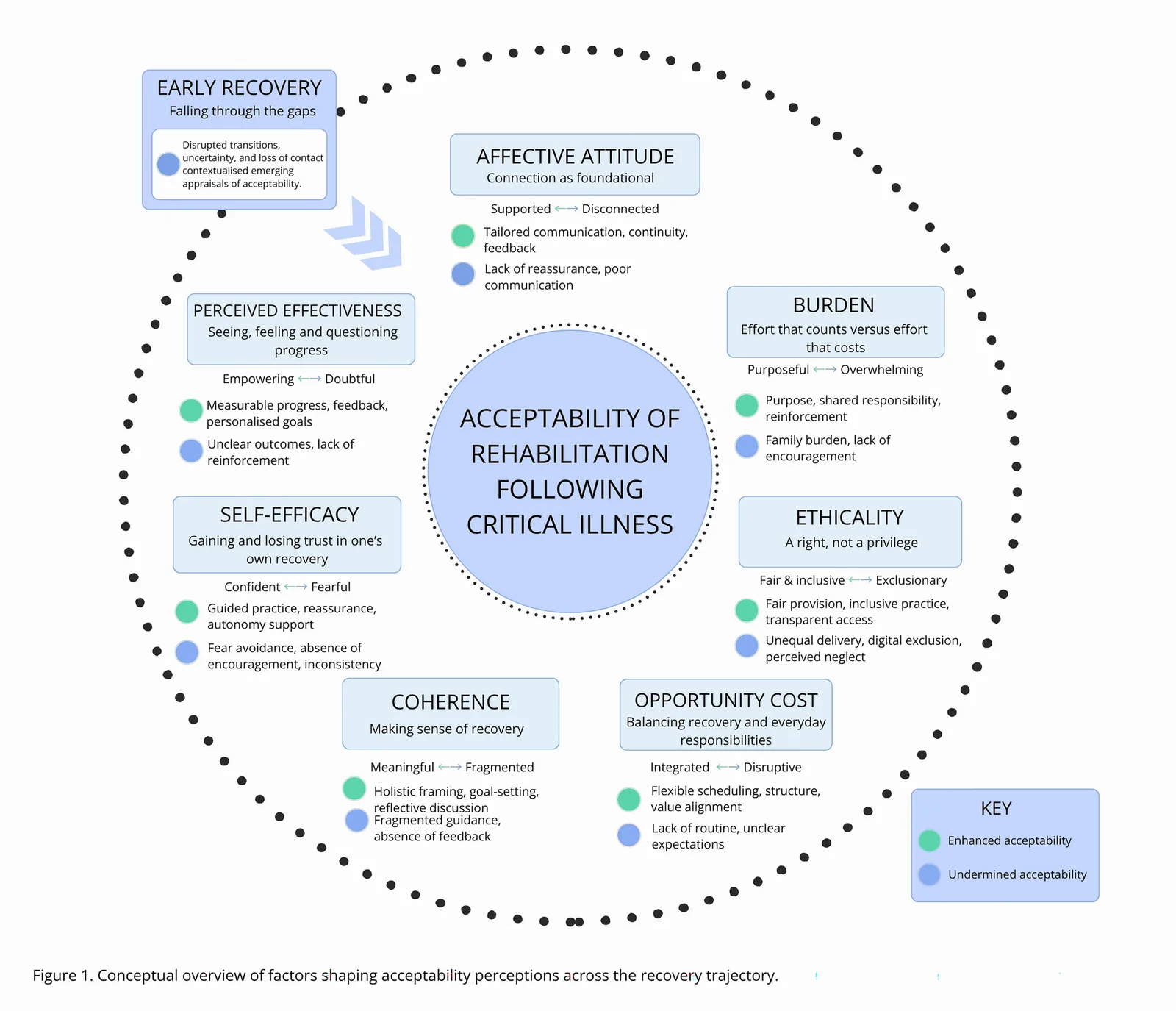
Conceptual overview of factors shaping acceptability perceptions across the recovery trajectory

A coding tree of the thematic structure is presented in Additional file 8, with expanded detail for each theme and subtheme provided in Additional file 9. Figure 1 provides an overview of these themes, illustrating how early recovery experiences and construct-based appraisals interacted to shape perceptions of acceptability across the recovery trajectory. Within each TFA domain, the figure presents factors that enhanced acceptability alongside factors that undermined or complicated acceptability. These were synthesised through comparison across remote rehabilitation, standard care and staff accounts, with each theme written as a continuum of more and less acceptable experiences rather than as uniformly positive or negative appraisals.

### Early recovery—falling through gaps in follow-up

Across both patient and staff groups, early recovery was described as a precarious transition following discharge, marked by a sudden withdrawal of professional support and limited guidance on how to recover. Participants recounted disorienting and isolating returns home, while staff emphasised the sharp drop-off in care and absence of structured follow-up. These experiences of uncertainty and perceived abandonment shaped expectations of support and, in turn, how the acceptability of rehabilitation and standard care was appraised, foregrounding the importance of continuity and reassurance within the recovery trajectory.

### Affective attitude—connection as foundational to recovery

As recovery progressed, participants’ accounts diverged, revealing emotional differences in how remote rehabilitation and standard care were experienced and valued. For those in the intervention, regular contact reinstated reassurance and motivation after a period of isolation, contributing to a more positive emotional appraisal of the support received. This contact helped transform the home from a place of vulnerability into one of the safety and continuity. Staff highlighted the importance of humanised, personalised interaction as central to fostering this sense of connection. In contrast, participants receiving standard care described how the absence of ongoing contact deepened uncertainty and eroded trust in their recovery, shaping less favourable emotional responses to the support available.

### Burden—effort that counts versus effort that costs

Recovery after ICU was universally effortful; however, the acceptability of remote rehabilitation and standard care was shaped by whether that effort felt meaningful or depleting. For those receiving remote rehabilitation, exertion was often framed as a purposeful investment that generated progress and motivation, supporting more positive appraisals of the effort required. Staff viewed this effort through a more protective lens, at times anticipating burden, where participants described determination while also recognising their own emotional involvement in supporting and containing distress. In contrast, participants receiving standard care described recovery as an ongoing struggle in which energy was expended rather than replenished, contributing to perceptions of effort as burdensome. Much of this hidden labour was absorbed by family members, whom participants felt carried both practical and emotional strain.

### Opportunity costs—balancing recovery with everyday responsibilities

As recovery became embedded in daily life, participants described weighing engagement with remote rehabilitation and standard care against personal and practical responsibilities. The acceptability of support was shaped by the extent to which it could be accommodated alongside everyday roles. For those receiving remote rehabilitation, recovery was deliberately prioritised and integrated into existing routines, with flexibility enabling participation without disrupting daily life. This supported more favourable appraisals of the demands placed on time and energy. Staff similarly viewed this adaptability as central to sustaining engagement, ensuring the programme complemented rather than competed with everyday responsibilities. In contrast, participants receiving standard care described fewer structured prompts to prioritise recovery, meaning it was more easily displaced by the demands of daily life, contributing to a lower perceived priority and reduced engagement.

### Ethicality—rehabilitation as a right, not a privilege

Across accounts, participants expressed a strong moral conviction that structured rehabilitation should be a right rather than a privilege, positioning acceptability in relation to fairness and equity across rehabilitation and standard care. Among those receiving remote rehabilitation, gratitude was often accompanied by discomfort that others were excluded from similar support, shaping a more ambivalent appraisal of its acceptability. Staff echoed this concern, viewing unequal access as inconsistent with the principles of equitable care. In contrast, participants in standard care experienced this inequity more directly, with some perceiving their needs as overlooked within a system of competing priorities, contributing to less favourable evaluations of the support available.

Digital access further shaped perceptions of fairness. While some viewed remote delivery as inclusive and adaptable, others experienced it as introducing new barriers, particularly for older or less technologically confident individuals, complicating perceptions of acceptability across both forms of support.

### Coherence—making sense of recovery

Participants’ ability to make sense of recovery shaped how acceptable remote rehabilitation and standard care were perceived to be, particularly where physical, psychological, and social aspects of recovery could be integrated into a coherent narrative. For those receiving remote rehabilitation, structured input, feedback, and visible markers of progress supported this sense-making process, contributing to clearer and more favourable appraisals of support. Staff reinforced this clarity through goal setting and reflective conversations that highlighted progress over time. In contrast, participants in standard care often struggled to find meaning or direction, describing frustration that psychological and emotional aspects of recovery were overlooked, which contributed to less coherent and less acceptable experiences of support. For some, peer support partially compensated for this gap by linking individual experiences into a shared narrative of recovery. However, where coherence was already supported through the structured one-to-one sessions offered within remote rehabilitation, the acceptability of peer support was more variable. While reassuring for some, others found it emotionally demanding, with shared struggles at times amplifying distress rather than easing it.

### Perceived effectiveness—seeing, feeling and questioning progress

Across accounts, participants evaluated the acceptability of remote rehabilitation and standard care in relation to whether they could see or feel progress. Tangible signs of improvement reassured them that support was effective, while the absence of such evidence left recovery feeling uncertain or incomplete. For those receiving remote rehabilitation, measurable milestones and consistent feedback strengthened these perceptions, with participants describing noticeable physical gains that restored motivation and hope. Staff similarly recognised the restorative value of remote rehabilitation, viewing it as instrumental in helping individuals rebuild strength and regain a sense of normality. In contrast, participants receiving standard care also sought evidence of progress but often found it harder to recognise, contributing to more tentative appraisals of effectiveness. Some described small but meaningful gains, while others experienced their improvement as partial or fragile, reinforcing uncertainty about whether recovery was progressing as expected*.*

### Self-efficacy—gaining and losing trust in one’s own recovery

Across accounts, the acceptability of remote rehabilitation and standard care was shaped by how confident participants felt in pushing themselves and how safe they felt doing so. Although all described testing limits and relearning trust in their bodies, confidence developed differently depending on the support available. For those receiving remote rehabilitation, reassurance and guided experimentation supported a growing sense of mastery and safety, contributing to more confident and positive appraisals of their recovery. In contrast, participants in standard care were often left to rebuild confidence independently, which at times led to greater hesitation and anxiety about overexertion or harm, shaping less acceptable experiences of support. Staff similarly viewed the restoration of self-trust as central to recovery, emphasising the importance of creating space for individuals to regain confidence in their own capabilities.

## Discussion

This study examined how ICU survivors and staff perceived the acceptability of a remote multicomponent rehabilitation programme versus standard care following discharge from critical care. Descriptive TFAQ findings indicated higher acceptability across TFA domains among participants receiving remote rehabilitation compared with those receiving standard care. Qualitative findings provided insight into how and why these differences emerged, showing that acceptability was shaped by whether support provided continuity, made recovery understandable, and supported confidence and progress. Drawing on the TFA [19], the findings show how acceptability constructs interact to shape how remote rehabilitation and standard care are appraised as acceptable or insufficient within the recovery context.

These findings also help refine interpretation of the main iRehab trial results. Although the trial did not demonstrate a statistically significant effect on the primary outcome, the intervention was associated with improvements in leg strength/exercise capacity, fatigue, anxiety, and acceptability at 8 weeks, and EQ-5D-5L utility score at 6 months [20]. Subgroup analysis found that participants who received mechanical ventilation for 7 days or less also had significantly improved quality of life, whereas those with mechanical ventilation for more than 7 days did not. The qualitative findings suggest that these outcomes may have been supported by features of remote rehabilitation that were largely absent from standard care, including structured guidance, regular professional contact, reassurance, feedback and opportunities to make sense of recovery. These features helped participants recognise progress and rebuild confidence, whereas standard care participants more often described uncertainty, fragmented follow-up and unsupported trial and error. While the qualitative data cannot explain the absence of statistically significant effects on depression or illness perceptions, they suggest that the perceived value of remote rehabilitation depended on the timing, flexibility, dose and fit of support with individual recovery contexts. Thus, this sub-study complements the main trial by identifying how remote rehabilitation was experienced as acceptable and useful while also demonstrating why usual care was often appraised as insufficient.

The iRehab trial was underpinned by a biopsychosocial model, consistent with broader approaches to post-ICU recovery [26, 27]. The findings suggest that recovery support is experienced as more acceptable when physical, psychological, and social aspects of recovery are addressed in a coordinated way. The structured, multicomponent nature of rehabilitation appeared to support this integration, enabling participants to engage with different aspects of recovery in a connected and manageable way while also rebuilding confidence in their post-ICU selves [28]. In contrast, standard care was often experienced as more fragmented, requiring individuals to navigate different aspects of recovery independently, which contributed to less favourable perceptions of support. In practice, this highlights the importance of rehabilitation models that embed coordinated biopsychosocial provision, like iRehab, [21] to promote both recovery and its acceptability.

The acceptability of support also appeared closely tied to how well individuals were supported to make sense of their recovery. This can be understood through a self-regulation lens, whereby individuals actively appraise symptoms, update their understanding of recovery, and adjust coping responses over time [29]. For those receiving remote rehabilitation, guided mastery and feedback made progress visible and effort meaningful, enabling participants to interpret their experiences with greater confidence and, in turn, experience support as more acceptable and worthwhile. In contrast, where such input was absent, individuals in standard care were more likely to rely on unsupported trial and error, which could reinforce uncertainty and contribute to less favourable perceptions of support. These findings align with behaviour change research showing that feedback and vicarious experience enhance self-efficacy, whereas unsupported self-management can erode it [30]. Clinically, these mechanisms translated into concrete practices: regular scheduled contact; personalised discussion of symptoms, concerns, and priorities; small, achievable goal setting; review of goals and progress at each contact; reassurance about safe progression; feedback that made improvements visible; and flexibility in the mode, pace, and content of support.

Connection also underpinned the acceptability of support through its influence on affective responses. Regular contact with professionals fostered reassurance and accountability, contributing to more positive evaluations of recovery support. In contrast, the absence of such contact in standard care left participants uncertain about their progress and more likely to question the adequacy of support. Consistent with evidence that empathic, person-centred communication enhances engagement and well-being [31] these findings suggest that relational continuity may be important in post-ICU recovery [32]. Ensuring consistent follow-up contact, whether through remote, hybrid, telephone, or community-based models, may help sustain reassurance and emotional safety during recovery.

Perceived burden was a key factor shaping acceptability, but this differed across intervention contexts. Amongst those receiving remote rehabilitation, effort was more often experienced as purposeful and scaffolded, meaning that demands were viewed as tolerable or even worthwhile. This is consistent with prehabilitation research, where structured activity is valued despite its intensity [33]. For those receiving standard care, hidden burdens were often absorbed by families, extending beyond patients themselves. This adds to prior work on carer strain after ICU [34] suggesting that family burden influences how acceptable rehabilitation feels to survivors. Interventions should, therefore, acknowledge and support family members to mitigate this hidden labour and enhance acceptability across the recovery dyad.

The findings also revealed that some components worked well for certain participants but were more challenging for others, depending on readiness and context. Peer support illustrates this. For many, it reduced isolation and helped to normalise experience, but for those who felt unready, it could be emotionally difficult [35, 36]. However, its optional nature allowed participants to engage when it felt appropriate, which enhanced acceptability. In contrast, participants in standard care often described a lack of follow-up and continuity, meaning that peer support, where accessed, was valued as a substitute for absent support. This suggests that peer support should be offered flexibly and optionally, with attention to timing, readiness, and format.

A similar pattern was seen with digital delivery. For many participants, remote access reduced burden and supported engagement, but for those with lower digital confidence it shifted additional effort onto participants who were less confident managing it. This duality reflects wider debates in digital health, where acceptability is shaped by individual capability and social context [37–39]. While emerging critical care telerehabilitation studies suggest that remote rehabilitation can be delivered in ICU and mechanically ventilated populations [40], our findings add nuance by showing that acceptability may depend on whether digital confidence, resources, and support needs are addressed during delivery. This may be particularly important for older, frailer, cognitively affected, or sensory-impaired post-ICU survivors, who may be less able to engage with remote platforms without additional support.

Importantly, participants in this study had already consented to take part in a remotely delivered clinical trial, and eligibility criteria required English proficiency and email access. This means the sample should be understood as reflecting those for whom remote trial participation was already feasible and acceptable at the point of enrollment, rather than the wider population of ICU survivors who may face greater barriers to digital access. Future research and implementation should, therefore, prioritise more inclusive recruitment approaches, alternative access routes, simplified interfaces, clear onboarding and training, proactive technical support, telephone-supported options, involvement of family carers or local care coordinators where appropriate, and hybrid models combining remote and face-to-face support. These strategies may help to ensure that remote rehabilitation does not widen existing inequalities in post-ICU recovery support.

### Strengths and limitations

This study has several strengths. Using the TFA to guide both data collection and analysis enabled a theory-informed yet flexible exploration of post-ICU rehabilitation. The combined inductive–deductive approach allowed unanticipated insights while maintaining conceptual coherence. The sample size aligned with recommendations for multisite qualitative research, [22] allowing for sufficient depth within groups and comparison across contexts while maintaining analytic manageability. Including both patient and staff perspectives offered a more comprehensive understanding of acceptability than studies limited to recipients alone, while the inclusion of both intervention and standard care participants enabled comparison across supported and routine recovery contexts. Embedding the qualitative work within a pragmatic trial further strengthened ecological validity by capturing experiences within real-world service constraints.

Several limitations should be noted. As this qualitative study was embedded within the iRehab trial, participants’ perceptions of whether the allocated support helped their recovery may have shaped both TFAQ responses and interview narratives. This is consistent with the focus of the study, as perceived effectiveness is a core construct within the TFA, but means that findings should be interpreted as evidence of perceived acceptability and perceived benefit rather than objective intervention effectiveness. The trial context, while enhancing real-world relevance, may also have constrained flexibility and shaped participant experiences. Standard care varied across sites and participants, which may have influenced acceptability ratings and interview accounts. However, this heterogeneity reflects real-world variation in post-ICU follow-up and rehabilitation provision, which the study sought to understand.

Despite purposive sampling, most participants were White and English-speaking, limiting transferability to more diverse populations. The remote rehabilitation and standard care interview samples also differed descriptively in age and employment status, with standard care participants being older on average and more likely to have been retired before ICU admission. These differences were not tested statistically, in keeping with the qualitative design of the sub-study, but may have shaped participants’ comparative appraisals, particularly in relation to digital confidence, daily roles, employment-related opportunity costs, and how recovery was prioritised within everyday life.

The transferability of these findings should also be considered in the context of international variation in post-ICU care pathways. As this was a UK-based study, the acceptability and perceived value of iRehab may partly reflect the organisation and accessibility of existing NHS rehabilitation services and follow-up care. Participants commonly described fragmented transitions from hospital to community services and limited access to structured rehabilitation, but healthcare systems differ substantially in how post-ICU rehabilitation is funded, organised and delivered, including through specialist rehabilitation services, community-based integrated care and insurance-funded models. Consequently, the experiences reported here may not be directly transferable to all healthcare settings. However, the importance of coordinated follow-up, continuity of care, flexible rehabilitation delivery and person-centred support is likely to remain relevant across different models of healthcare delivery.

Staff interviews were limited to those delivering the intervention, excluding system-level perspectives. Informal carers were not directly interviewed, despite participants and staff describing families as important sources of practical and emotional support during recovery. The findings relating to family burden should, therefore, be interpreted as patient and staff perspectives on carer involvement, rather than direct evidence of carers’ own experiences. Future research should examine dyadic patient–carer perspectives on the acceptability and burden of post-ICU rehabilitation. The views of those who declined participation also remain unknown.

## Conclusions

Acceptability of recovery support was shaped by how meaningful, manageable, and responsive care felt in the context of post-ICU recovery. Rehabilitation was generally described as acceptable, as it provided structure, continuity, and support for confidence-building and progress, whereas standard care was often experienced as lacking these features. Acceptability varied across individuals, with components such as peer support and digital delivery valued by many but not by all, depending on readiness, context, and capability. These findings highlight the need for flexible, coordinated rehabilitation pathways that can respond to differing needs while ensuring access to meaningful support.

## Supplementary Information

Supplementary material 1.

## Data Availability

The datasets generated and/or analysed during the current study are not publicly available due to the sensitive and potentially identifiable nature of qualitative data but are available from the corresponding author on reasonable request.

## Abbreviations

ICU: Intensive Care Unit
TFA: Theoretical Framework of Acceptability
TFAQ: Theoretical Framework of Acceptability Questionnaire
RCT: Randomised Controlled Trial
RR: Remote rehabilitation
SC: Standard care

## References

[1] ICNARC. Case Mix Programme Summary Statistics 2019–20. London: Intensive Care National Audit & Research Centre; 2020. https://www.icnarc.org/reports/case-mix-programme-summary-statistics-2019-20

[2] Bose S, Hoenig B, Karamourtopoulos M, Banner-Goodspeed V, Brown S. Beyond survival: identifying what matters to survivors of critical illness. Crit Care. 2021;25(1):129. 10.1186/s13054-021-03565-x. 33823888 10.1186/s13054-021-03565-x PMC8025480

[3] Brown SM, Bose S, Banner-Goodspeed V, Beesley SJ, Dinglas VD, Hopkins RO, et al. Approaches to addressing post-intensive care syndrome among intensive care unit survivors. A narrative review. Ann Am Thorac Soc. 2019;16(8):947–56. 10.1513/annalsats.201812-913fr. 31162935 10.1513/AnnalsATS.201812-913FR

[4] Hopkins RO, Suchyta MR, Kamdar BB, Darowski E, Jackson JC, Needham DM. Instrumental activities of daily living after critical illness: a systematic review. Ann Am Thorac Soc. 2017;14(8):1332–43. 10.1513/annalsats.201701-059sr. 28463657 10.1513/AnnalsATS.201701-059SR PMC5566273

[5] Zhang Z, Yang L, Cao H. The interactivity and independence of recovery challenges and coping strategies for ICU survivors and their caregivers: a systematic review and meta-synthesis. BMC Nurs. 2024;23(1):895. 10.1186/s12912-024-02542-3. 39695626 10.1186/s12912-024-02542-3 PMC11656989

[6] Porter LL, Wijntjes K, Simons KS, van den Boogaard M, Custers JA, Zegers M. Beyond functional outcomes: exploring quality of life after critical illness—a qualitative study. Crit Care Med. 2025;53(6):e1190–201. 10.1097/ccm.0000000000006665. 40163331 10.1097/CCM.0000000000006665 PMC12124206

[7] Hill AD, Fowler RA, Pinto R, Herridge MS, Cuthbertson BH, Scales DC. Long-term outcomes and healthcare utilization following critical illness-a population-based study. Crit Care. 2016;20(1):76. 10.1186/s13054-016-1248-y. 27037030 10.1186/s13054-016-1248-y PMC4818427

[8] Lone NI, Lee R, Salisbury L, Donaghy E, Ramsay P, Rattray J, et al. Predicting risk of unplanned hospital readmission in survivors of critical illness: a population-level cohort study. Thorax. 2019;74(11):1046–54. 10.1136/thoraxjnl-2017-210822. 29622692 10.1136/thoraxjnl-2017-210822

[9] Griffiths J, Hatch RA, Bishop J, Morgan K, Jenkinson C, Cuthbertson BH, et al. An exploration of social and economic outcome and associated health-related quality of life after critical illness in general intensive care unit survivors: a 12-month follow-up study. Crit Care. 2013;17(3):R100. 10.1186/cc12745. 23714692 10.1186/cc12745 PMC3706775

[10] Kamdar BB, Huang M, Dinglas VD, Colantuoni E, Von Wachter TM, Hopkins RO, et al. Joblessness and lost earnings after acute respiratory distress syndrome in a 1-year national multicenter study. Am J Respir Crit Care Med. 2017;196(8):1012–20. 10.1164/rccm.201611-2327OC. 28448162 10.1164/rccm.201611-2327OC PMC5649982

[11] Connolly B, Douiri A, Steier J, Moxham J, Denehy L, Hart N. A UK survey of rehabilitation following critical illness: implementation of NICE Clinical Guidance 83 (CG83) following hospital discharge. BMJ Open. 2014;4(5):e004963. 10.1136/bmjopen-2014-004963. 24833691 10.1136/bmjopen-2014-004963 PMC4025447

[12] Hawley-Hague H, Lasrado R, Martinez E, Stanmore E, Tyson S. A scoping review of the feasibility, acceptability, and effects of physiotherapy delivered remotely. Disabil Rehabil. 2023;45(23):3961–77. 10.1080/09638288.2022.2138574. 36325612 10.1080/09638288.2022.2138574

[13] Goldman JG, Merkitch D, Brewington D, Peirce H, Rho M, Jayabalan P, et al. Patient experiences receiving rehabilitation care via telehealth: identifying opportunities for remote care. Front Rehabil Sci. 2023;4:1049554. 10.3389/fresc.2023.1049554. 36817717 10.3389/fresc.2023.1049554 PMC9932031

[14] Edwards D, Williams J, Carrier J, Davies J. Technologies used to facilitate remote rehabilitation of adults with deconditioning, musculoskeletal conditions, stroke, or traumatic brain injury: an umbrella review. JBI Evid Synth. 2022;20(8):1927–68. 10.11124/jbies-21-00241. 35971198 10.11124/JBIES-21-00241

[15] Porter LL, Simons KS, Turnbull AE, Corsten S, Westerhof B, Rettig TC, et al. Discrepancy between functional outcomes and perceived health post-intensive care unit: a prospective cohort study. Ann Am Thorac Soc. 2025;22(2):255–62. 10.1513/annalsats.202405-564oc. 39441100 10.1513/AnnalsATS.202405-564OC

[16] Kean S, Salisbury LG, Rattray J, Walsh TS, Huby G, Ramsay P. ‘Intensive care unit survivorship’-a constructivist grounded theory of surviving critical illness. J Clin Nurs. 2017;26(19–20):3111–24. 10.1111/jocn.13659. 27875013 10.1111/jocn.13659

[17] O’Neill B, Green N, Blackwood B, McAuley D, Moran F, MacCormac N, et al. Recovery following discharge from intensive care: what do patients think is helpful and what services are missing? PLoS ONE. 2024;19(3):e0297012. 10.1371/journal.pone.0297012. 38498470 10.1371/journal.pone.0297012 PMC10947670

[18] Parry SM, Knight LD, Connolly B, Baldwin C, Puthucheary Z, Morris P, et al. Factors influencing physical activity and rehabilitation in survivors of critical illness: a systematic review of quantitative and qualitative studies. Intensive Care Med. 2017;43(4):531–42. 10.1007/s00134-017-4685-4. 28210771 10.1007/s00134-017-4685-4

[19] Sekhon M, Cartwright M, Francis JJ. Acceptability of healthcare interventions: an overview of reviews and development of a theoretical framework. BMC Health Serv Res. 2017;17(1):88. 10.1186/s12913-017-2031-8. 28126032 10.1186/s12913-017-2031-8 PMC5267473

[20] O’Neill B, Bradley JM, Connolly B, et al. Remote multicomponent rehabilitation in intensive care unit survivors: a randomized clinical trial. JAMA. 2026. 10.1001/JAMA.2026.7401. 42150121 10.1001/jama.2026.7401 PMC13184783

[21] O’Neill B, Bradley JM, Connolly B, et al. Remote multicomponent rehabilitation compared to standard care for survivors of critical illness after hospital discharge (iRehab): a protocol for a randomised controlled assessor-blind clinical and cost-effectiveness trial. NIHR Open Res. 2025;5:29. 10.3310/NIHROPENRES.13910.2. 40443419 10.3310/nihropenres.13910.2 PMC12120417

[22] Gale NK, Heath G, Cameron E, Rashid S, Redwood S. Using the framework method for the analysis of qualitative data in multi-disciplinary health research. BMC Med Res Methodol. 2013;13(1):117. 10.1186/1471-2288-13-117. 24047204 10.1186/1471-2288-13-117 PMC3848812

[23] Saunders B, Sim J, Kingstone T, Baker S, Waterfield J, Bartlam B, et al. Saturation in qualitative research: exploring its conceptualization and operationalization. Qual Quant. 2018;52(4):1893–907. 10.1007/s11135-017-0574-8. 29937585 10.1007/s11135-017-0574-8 PMC5993836

[24] Sekhon M, Cartwright M, Francis JJ. Development of a theory-informed questionnaire to assess the acceptability of healthcare interventions. BMC Health Serv Res. 2022;22(1):279. 10.1186/s12913-022-07577-3. 35232455 10.1186/s12913-022-07577-3 PMC8887649

[25] Paynter C, McDonald C, Story D, Francis JJ. Application of the theoretical framework of acceptability in a surgical setting: theoretical and methodological insights. Br J Health Psychol. 2023;28(4):1153–68. 10.1111/bjhp.12677. 37353989 10.1111/bjhp.12677

[26] Üstün TB, Chatterji S, Bickenbach J, Kostanjsek N, Schneider M. The International Classification of Functioning, Disability and Health: a new tool for understanding disability and health. Disabil Rehabil. 2003;25(11–12):565–71. 10.1080/0963828031000137063. 12959329 10.1080/0963828031000137063

[27] Stucky K, Warren AM. Rehabilitation psychologists in critical care settings. In: Brenner LA, Reid-Arndt SA, Elliott TR, Frank RG, Caplan B, editors. Handbook of rehabilitation psychology. 3rd ed. Washington, DC: American Psychological Association; 2019. p. 483–96. 10.1037/0000129-029.

[28] Ewens BA, Hendricks JM, Sundin D. Surviving ICU: stories of recovery. J Adv Nurs. 2018;74(7):1554–63. 10.1111/jan.13556. 29489028 10.1111/jan.13556

[29] Leventhal H, Phillips LA, Burns E. The Common-Sense Model of Self-Regulation (CSM): a dynamic framework for understanding illness self-management. J Behav Med. 2016;39(6):935–46. 10.1007/s10865-016-9782-2. 27515801 10.1007/s10865-016-9782-2

[30] Ashford S, Edmunds J, French DP. What is the best way to change self‐efficacy to promote lifestyle and recreational physical activity? A systematic review with meta‐analysis. Br J Health Psychol. 2010;15(2):265–88. 10.1348/135910709x461752. 19586583 10.1348/135910709X461752

[31] Howick J, Moscrop A, Mebius A, Fanshawe TR, Lewith G, Bishop FL, et al. Effects of empathic and positive communication in healthcare consultations: a systematic review and meta-analysis. J R Soc Med. 2018;111(7):240–52. 10.1177/0141076818769477. 29672201 10.1177/0141076818769477 PMC6047264

[32] Hudson BF, Best S, Stone P, Noble TB. Impact of informational and relational continuity for people with palliative care needs: a mixed methods rapid review. BMJ Open. 2019;9(5):e027323. 10.1136/bmjopen-2018-027323. 31147362 10.1136/bmjopen-2018-027323 PMC6549611

[33] Smyth E, Brennan L, Enright R, Sekhon M, Dickson J, Hussey J, et al. The acceptability of exercise prehabilitation before cancer surgery among patients, family members and health professionals: a mixed methods evaluation. Support Care Cancer. 2024;32(6):399. 10.1007/s00520-024-08574-4. 38819477 10.1007/s00520-024-08574-4 PMC11142941

[34] van Beusekom I, Bakhshi-Raiez F, de Keizer NF, Dongelmans DA, van der Schaaf M. Reported burden on informal caregivers of ICU survivors: a literature review. Crit Care. 2015;20(1):16. 10.1186/s13054-016-1185-9. 10.1186/s13054-016-1185-9 PMC4721206 26792081

[35] Wasilewski MB, Rios J, Simpson R, Hitzig SL, Gotlib Conn L, MacKay C, et al. Peer support for traumatic injury survivors: a scoping review. Disabil Rehabil. 2023;45(13):2199–232. 10.1080/09638288.2022.2083702. 35680385 10.1080/09638288.2022.2083702

[36] Mikolajczak-Degrauwe K, Slimmen SR, Gillissen D, de Bil P, Bosmans V, Keemink C, et al. Strengths, weaknesses, opportunities and threats of peer support among disadvantaged groups: a rapid scoping review. Int J Nurs Sci. 2023;10(4):587–601. 10.1016/j.ijnss.2023.09.002. 38020843 10.1016/j.ijnss.2023.09.002 PMC10667317

[37] Perski O, Short CE. Acceptability of digital health interventions: embracing the complexity. Transl Behav Med. 2021;11(7):1473–80. 10.1093/tbm/ibab048. 33963864 10.1093/tbm/ibab048 PMC8320880

[38] Kenny E, Byrne M, McEvoy JW, Connolly S, McSharry J. Exploring patient experiences of participating in digital cardiac rehabilitation: a qualitative study. Br J Health Psychol. 2024;29(1):149–64. 10.1111/bjhp.12692. 37722874 10.1111/bjhp.12692

[39] Morton K, Dennison L, May C, Murray E, Little P, McManus RJ, et al. Using digital interventions for self-management of chronic physical health conditions: a meta-ethnography review of published studies. Patient Educ Couns. 2017;100(4):616–35. 10.1016/j.pec.2016.10.019. 28029572 10.1016/j.pec.2016.10.019 PMC5380218

[40] Rosa RG, Moraes RB, Trott G, et al. Integrated telehealth rehabilitation and quality of life in mechanically ventilated adults: a randomized clinical trial. JAMA. 2026. 10.1001/JAMA.2026.10617. 42268591 10.1001/jama.2026.10617 PMC13254807

