# Supplementary material for: Factors influencing the acceptability of remote multicomponent rehabilitation versus standard care following critical illness: a theory-informed qualitative study embedded within the iRehab trial
Source: J Intensive Care. 2026 Jul 26;14:81. doi: 10.1186/s40560-026-00908-0 (PMC13504833; doi:10.1186/s40560-026-00908-0)
Supplement: Supplementary file 1 [file 40560_2026_908_MOESM1_ESM.docx]

**Factors influencing the acceptability of remote multicomponent rehabilitation versus standard care following critical illness: a theory-informed qualitative study embedded within the iRehab trial**

Supplementary Material

**Table of Contents**

Additional file 1: COREQ checklist

Additional file 2: Interview guide (patients)

Additional file 3: Interview guide (staff)

Additional file 4: Modified TFAQ (intervention)

Additional file 5: Modified TFAQ (standard care)

Additional file 6: Modified TFAQ (staff)

Additional file 7: Analytical procedures

Additional file 8: Coding tree

Additional file 9: Overview of themes, subthemes and quotations

Additional file 10: iRehab trial investigators

Additional file 11: Acknowledgements

## Additional file 1: COREQ checklist

## Additional file 2: Interview guide (Patients)

**Remote multicomponent rehabilitation in survivors of critical illness after hospital discharge – the iRehab Trial**

**Semi-Structured Participant Interview Schedule – Rehabilitation programme group**

**Introduction**

**INTRODUCTIONS AND PURPOSE:** e.g. you are involved in the remote rehabilitation after ICU trial (iRehab). The trial is exploring the effectiveness of a support and exercise programme (the iRehab programme) compared to usual care for people recovering after intensive care. The purpose of this discussion is to find out in more detail about your views and experience of being involved in the iRehab trial. We have a short list of topics that we will use to help guide our discussion. There are no right or wrong answers.

**CONFIRM CONSENT VERBALLY:** e.g. you may remember you were given an information sheet about taking part in an interview with a researcher and gave your consent for this. Our interview will last up to one hour (depending on how much you have to say) and will be recorded. You can stop the interview at any time. The content will be strictly confidential and once the recording is typed up then the recording will be deleted. **Your name will not appear with the recording or any of the material relating to this study.** Are you happy to begin?

START OF RECORDING:

**Consent:** Interviewer reads aloud the patient interview consent form and records responses. If participant does not provide consent, end call. END RECORDING.

START NEW RECORDING:

1. **Experiences after discharge from hospital**

- How are you feeling now? (Probe for examples of things they can/ can’t do/ would like to do?)
- How were you feeling when you were discharged from hospital? *(Probe for differences in physical health, mental health, daily self-care, social activities/ other responsibilities.)*
- How would you describe your recovery up to this point? *(Probe for comparison with expectation of recovery)*
- Is there anything in particular that has helped with your recovery? *(if mention iRehab, take initial responses, and explain these will be followed up in more detail).*
- Did you receive any advice/ support/ care after you were discharged from hospital?
- Did you feel you needed advice/support/ care to help with your recovery after discharge from hospital? Please explain your answer.

2. **Experiences joining the iRehab study**

- Do you remember when you first heard about the study? What were your first impressions of the study?
- When is a good time to talk to people about the study?
- How did you join the study?
- Can you explain to me what the study is about? Why is it being done?
- What were your expectations about joining the study?
- When/ how did you find out which group you were going to join? How was this decided? How did you feel about this?
- What happened when you joined the study? (e.g. questionnaires, sit to stand test). Any advice to improve your experience of joining the study?

***Standard care participants, go to Section 4.***

***Section 3: Feedback on the iRehab Programme (iRehab Participants only)***

**Participant’s feedback on the iRehab programme:**

***(Reflect back on the weekly iRehab programme)***

1. **How do you feel that the iRehab programme has affected your health?**

- physical health/ functional
- mental health
- other (social)
- difference in health now compared to just before the programme started

2. **Do you think your relatives/carers/friends see a difference in you?**

- differences in physical health
- mental health/functional
- other (social)

General feedback on the iRehab Programme

3. **How did you find the iRehab programme overall:**

- What did you spend most time doing? What was most/ least helpful? Likes/ Dislikes
- How engaged would you say you were with the programme? Did you do any activities outside the online/ phone appointments?
- What are the key things you got out of the programme?
- Was it beneficial for you (explain)? Other people? Would you recommend the programme to others?
- Anything you would change about the programme?
- How would you describe iRehab to a patient who was discharged from intensive care?
- iRehab programme is a described as ‘remote’ support and rehabilitation programme? What does that mean to you? Was it clear? What are the benefits of remote support? Any drawbacks/ limitations?

*The iRehab programme is a remote rehabilitation programme that you can access from home. It is available to people over a six-week period. The programme is tailored to individual needs. It has four components: weekly one-to-one appointments to chat about any problems and do some exercises; online exercise sessions; group support sessions to share experiences with other patients recovering after intensive care; a manual with advice to manage symptoms.*

- Is that a good description of the programme? Explain your answer.
- Did the programme meet your expectations? Explain your answer.

Specific feedback on the iRehab programme:

*(Let me talk more about the iRehab programme, reflect back)*

4. **How easy did you find about the whole iRehab programme (one to one, exercise/group exercise/support group)?**

- Feel able to do the programme
- Understand how to do it
- Use of remote delivery
- Did you find the programme motivating? What helped you keep going with it/ or did you keep going?
- What about when you were tempted not to do anything
- Were there any difficulties with taking part?
- How did you mainly access the programme? (online – computer/ smart phone /mobile/ landline phone/ manual). Any technical issues?
- Any issues with language/ other problems taking part?

5. **What did you think of your one-to-one calls with your iRehab specialist?**

- How many times did you speak to specialist (too much, too little, about right?) Length of sessions? /. The programme involves 6 weekly appointments (too much, too little, about right?) Any suggestions to improve organisation of sessions?
- What did you do during the sessions?
- How did you feel after the sessions? (Probe for any benefit physically/ emotionally?)
- Content - tailored to needs
- Information provided *(probe: is there additional education or information that you think would be useful? If yes probe examples of this)*
- Delivery (online /phone)
- Did you find it useful setting weekly goals?
- How about the overall goal, did you find this useful?
- Did you discuss any symptoms with your therapist? How helpful were the sessions to manage your symptoms?
- Which parts of the one-to-one appointments were you most satisfied with?
- Anything you would like to change about the one-to-one sessions?
- Were one-to-one sessions worthwhile/ helpful for your recovery? In what way? If no, why not?

6. **How useful were the supporting materials/manuals?**

- Action / coping plan / diary
- iRehab Recovery Pack
- iRehab Exercise Manual
- Step counter (if utilised)
  - How did the step-counter help you?

7. **How did you feel about the exercise part of the programme?**

- Individual and/or group exercise
- Whether level of fitness/ability was considered
- Additional probes: ease of following instructions/ demos; additional exercise completed outside set sessions (videos, manual, walking, other physical activity etc.); self-rated progress; self-rated engagement with exercise component; anything to help motivation/ suggestions to improve exercise component.
- Overall, did you feel you benefited from the exercise part of the programme? Explain.

8. **What are your thoughts about the online support group (iRehab café)?**

- How would you describe the sessions?
- Frequency of attendance and reason?
- Were there specific discussions that you liked/disliked?

Did you share things in the group? Why/ why not? Helpful to share/ listen?

9. **Overall, what was best about the programme for your recovery? (probe weekly appointments; online exercise sessions; iRehab café). Anything that you think was not helpful for your recovery?**

Views about the study questionnaires and fitness test:

10. **You completed a number of questionnaires and a short fitness test online or with the researcher. How did you find these?**

- Easy/difficult / like/didn’t like
- Comments on any measure in particular
- How suitable are the measures for someone recovering after intensive care?
- Is there anything else that would be important to you to record your progress/ recovery?
- Did you feel your taking part in the study was important? Explain your answer.

Continuing rehabilitation and recovery

11. **How confident are you that you could continue your recovery now that the programme has finished e.g. continue exercise, activity or other strategies**

- Are there any specific aspects from the programme that think will be helpful?
- What do you think would help you?

Conclusion:

12. **Did the iRehab programme speed up your recovery? Explain your answer.**
13. **What do you think might be the results of the iRehab trial?** (Explain study design to compare people’s health at 8 weeks between those who took part in the intervention and those who received standard NHS care). Explain your answer.
14. **Are you interested in the results of the study.** Please explain.
15. **What suggestions if any, would you give to improve the iRehab programme?**

- Would you recommend the iRehab programme to anyone else who has been in Intensive Care?
- Probe reasons

16. **Is there anything else that you would like to add regarding your experiences of taking part in the iRehab programme?**

**For Standard Care patients only**

Patient’s strategies for recovery and NHS standard care

1. **Can you tell me about anything you have done to help your recovery since coming home from hospital?** Probe for any activities to help with physical recovery, mental well-being. Has anything been helpful to speed up your recovery?

- Have you talked to anyone about your experiences in hospital / afterwards? Has this been helpful?
- Have you received any support/ advice from other people?
- Have you researched anything online (videos, articles, books) or elsewhere?
- Thinking about any of the activities you mentioned, has anything made a difference?
- Did hearing about the iRehab study prompt you to do anything to help your recovery?
- Did you have any follow-up contact with your NHS hospital following discharge? Can you explain further.

2. **Overall, what difference if any, do you think the activities you engaged with helped to speed up your recovery. Explain your answer.**

Participant’s feedback on the iRehab study/ programme:

3. **What did you do to take part in the study?**

- How did you find completing the questionnaires, online fitness test with the researcher? (ease/ difficulty/ like/ didn’t like).
- How suitable are the measures for someone recovering after intensive care?
- Is there anything else that would be important to you, to record your progress/ recovery?
- Did you have contact with anyone from the trial during follow-up?
- Did you feel your taking part in the study was important? Explain your answer.
- Can you remember anything about the iRehab programme? What did you think about it?

The iRehab programme is described as a ‘remote’ support and rehabilitation programme. Was it clear to you what that means?

*The iRehab programme is a remote rehabilitation programme that you can access from home. It is available to people over a six-week period. The programme is tailored to individual needs. It has four components: weekly one-to-one appointments to chat about any problems and do some exercises; online exercise sessions; group support sessions to share experiences with other patients recovering after intensive care; a manual with advice to manage symptoms.*

- Do you think you would find this helpful/ speed up your recovery? Probe what and why.

Continuing rehabilitation and recovery

4. **Is there anything you are doing now to help your recovery? Will you continue with it for the foreseeable future?**

- Are there any specific aspects from the programme that think will be helpful?
- What do you think would help you?

Conclusion:

5. **Have you done anything that helped speed up your recovery? What worked? Why?**
6. **What do you think might be the results of the iRehab trial?** (Explain study design to compare people’s health at 8 weeks between those who took part in the intervention and those who received standard NHS care). Please explain your answer.
7. **Are you interested in the results of the study.** Please explain.
8. **Would you recommend the study to others discharged from intensive care?** Why/ why not?
9. **What suggestions if any, would you have to improve the study?**
10. **Is there anything else that you would like to add regarding your experiences of the iRehab study?**

## Additional file 3: Interview guide (Staff)

**Semi-Structured Interview Schedule – Trial Staff/ Healthcare Professionals**

**Introduction**

**INTRODUCTIONS AND PURPOSE:** e.g. you are involved in the remote rehabilitation after ICU trial (iRehab). The trial is exploring the effectiveness of a support and exercise programme (the iRehab intervention) compared to usual care for people recovering after intensive care. The purpose of this discussion is to find out in more detail about your views and experience of being involved in the iRehab trial/ rehabilitation services. We have a short list of topics that we will use to help guide our discussion. There are no right or wrong answers.

**CONFIRM CONSENT VERBALLY:** e.g. you may remember you were given an information sheet about the iRehab interviews, and gave your consent for this. Our interview will last around **20-30 minutes**, and will be recorded. The content will be strictly confidential and once the recording is transcribed (typed up) then the recording will be deleted. **Your name will not appear with the recording or any of the material relating to this study.** Are you happy to begin?

NB: The questions asked will be asked as appropriate to the role of the healthcare professional/ staff member involvement in the trial/ rehabilitation service provision.

**Interview:**

START OF RECORDING

*Intervention staff*

Staff perceptions of being involved in the iRehab trial/intervention:

1. **We would you like to talk about what it was like for you being involved in the iRehab trial/ delivering the intervention to people discharged home after being in intensive care.**

- Have you cared for these types of patients before taking part in the iRehab trial?
- Have you taken part in delivering a trial/similar intervention before?
- Can you tell us a little about your role/involvement in the trial?

Ease of delivery of trial/intervention procedures

2. **What was it like carrying out your role in the trial?**

- How did you feel about describing the trial to others e.g. participants and their families?
- Was there anything tricky or difficult for you in delivering the iRehab trial/intervention? Please expand on those aspects you found either difficult or easy to deliver.
- Was there anything that helped you to deliver the trial/intervention?

3. **What was it like for you working with these participants who had been ventilated in ICU?**

- Did you find anything upsetting at all? e.g. do you think that therapists would require any additional or emotional support?
- Are there any tips for success that you think it would be important to share with other staff?

Intervention delivery [INTERVENTION TEAM ONLY]

*(Let’s talk more about the intervention, reflect back)*

4. **What are your thoughts on the content /components of the iRehab intervention itself? [INTERVENTION TEAM ONLY]**

- How did you feel about the content /components of the iRehab intervention?
- Were there specific parts or components that seemed particularly helpful to participants?
- Is there anything you would want to change about the content of intervention?
- Do you think the intervention was effective in supporting you to help patients achieve their goals?
- Did you think the participants taking part found the materials helpful?
- Is there anything about the participant materials you would want to change?
- Is there anything about the therapist materials you would want to change?

Trial/intervention training

5. **Did you receive enough/the right training to deliver the trial/ intervention? []**

- What aspects of the trial/intervention training did you find helpful?
- How easy/difficult was it to incorporate the learning and skills into the trial/intervention?
- Were there some strategies you found more helpful than others?

Acceptability of the intervention and future implementation

6. **What do you perceive are the of challenges of delivering the iRehab intervention in clinical practice?**

- Thinking about the future, if the iRehab intervention is found to be better than usual care, do you think it could be implemented across the NHS?
- In your opinion, how could implementation be best achieved? Is there anything which in your opinion would facilitate a widespread implementation?
- Thinking about the future, if the iRehab intervention is shown **not** to be better than usual care, how will you feel?

Concluding topic

7. **Is there anything else that you would like to add regarding your experiences of taking part in the trial/intervention?**

**Thank you for taking the time to take part in this interview**

*Trial staff/ health care professionals:*

Background

1. **Can you tell me about your professional background and involvement in the iRehab study?**

**For recruiters:**

- Have you previous experience of trial recruitment?
- How is your research team set up to recruit patients to ICU studies?
- How did you organise recruitment to iRehab?
- How is recruitment going? (probe for any difficulties, with screening, eligibility checks, approaching patients, decliners, trial enrolment processes)?
- Which patients are suitable for the iRehab study?
- Any not suitable? Any patients not recruited to iRehab (e.g. *language, technology, capability, co-morbidities)*
- What do you think about the inclusion/ exclusion criteria for the trial? Did you find these easy to apply?
- Did you co-enrol to iRehab? Can you explain more about your experiences of co-enrolment.
- What is going well about recruitment/ any challenges?
- Any barriers to recruitment?
- Was the PI involved in recruitment?
- How have patients responded to the trial and the intervention arm?
- Do patients have preferences? Have you discussed preferences? How do you feel about exploring preferences?
- Have you recruited patients from follow-up clinics? Explain your answer?

Rehabilitation services and the iRehab trial (if relevant)

2. **What needs do patients have when they are discharged from hospital?**

- *Are these needs being met? How? If not, why not?*
- What if anything can be done to speed their recovery/ quality of patient’s recovery?
- *What do you think about current NHS standard of care? Does it vary across the UK?*
- *Do you think there is a role for remote rehabilitation services in the UK? Why, why not?*
- *What do you think about the iRehab research question? Is it important? Explain your answer.*
- Would you say you have clinical equipoise? What does that mean?
- What do you think about the iRehab intervention?

*Prompt if necessary: The iRehab programme is a remote rehabilitation programme that patients can access from home. It is available to people over a six-week period. The programme is tailored to individual needs. It has four components: weekly one-to-one appointments to chat about any problems and do some exercises; online exercise sessions; group support sessions to share experiences with other patients recovering after intensive care; a manual with advice to manage symptoms.*

- What is standard care at your hospital? Are patients provided any follow-up services following discharge from ICU? Has that changed over the duration of the trial?
- Do you think iRehab might help people recover better/ faster? Explain your answer?
- Which aspects of the programme are most important?
- What do you think the outcome of the trial will be?

Acceptability of the intervention and future implementation (if relevant)

3. **What do you perceive are the of challenges of delivering the iRehab intervention in clinical practice?**

- Thinking about the future, if the iRehab intervention is found to be better than usual care, do you think it could be implemented across the NHS?
- In your opinion, how could implementation be best achieved? Is there anything which in your opinion would facilitate a widespread implementation?
- Thinking about the future, if the iRehab intervention is shown **not** to be better than usual care, how will you feel?

Concluding topic (all)

4. **Is there anything else that you would like to add regarding your experiences of taking part in the trial/intervention? [DELETE AS APPROPRIATE]**

- Are there any areas where trial processes could be improved?
- What was most challenging about your role? (e.g. probe for recruitment, intervention delivery, trial set up, retention, trial processes, blinding).
- Were you able to deliver the study as per protocol? What challenges were there?
- If you were doing the trial again, what would you do differently/ what have you learnt?

**Thank you for taking the time to take part in this interview**

END OF RECORDING

## Additional file 4: Modified TFAQ (Remote rehabilitation group)

You have received this questionnaire as you received the iREHAB programme as part of the iREHAB study. We are seeking to gain additional information about your opinion of the iREHAB programme. There are 8 questions which will take you approximately 5 minutes to complete. For each question, select the number on the 5-point scale that most accurately reflects your opinion. There are no right or wrong answers. Please ensure to submit the form once completed.

| **Did you like or dislike your iREHAB programme?** | **Strongly dislike** | **Dislike** | **No opinion** | **Like** | **Strongly like** |
| --- | --- | --- | --- | --- | --- |
|  | **1** | **2** | **3** | **4** | **5** |
|  |  |  |  |  |  |
| **How much effort did it take you to participate in your iREHAB programme?** | **No effort at all** | **A little effort** | **No opinion** | **A lot of effort** | **Huge effort** |
|  | **1** | **2** | **3** | **4** | **5** |
|  |  |  |  |  |  |
| **How fair is the iREHAB programme for people following and/or recovering from critical illness?** | **Very unfair** | **Unfair** | **No opinion** | **Fair** | **Very Fair** |
|  | **1** | **2** | **3** | **4** | **5** |
|  |  |  |  |  |  |
| **The iREHAB programme has improved your quality of life.** | **Strongly disagree** | **Disagree** | **No opinion** | **Agree** | **Strongly agree** |
|  | **1** | **2** | **3** | **4** | **5** |
|  |  |  |  |  |  |
| **It is clear to me how the iREHAB programme will help improve my quality of life** | **Strongly disagree** | **Disagree** | **No opinion** | **Agree** | **Strongly agree** |
|  | **1** | **2** | **3** | **4** | **5** |
|  |  |  |  |  |  |
| **How confident did you feel about participating in your iREHAB programme?** | **Very unconfident** | **Unconfident** | **No opinion** | **Confident** | **Very confident** |
|  | **1** | **2** | **3** | **4** | **5** |
|  |  |  |  |  |  |
| **The iREHAB programme interfered with my other priorities.** | **Strongly disagree** | **Disagree** | **No opinion** | **Agree** | **Strongly agree** |
|  | **1** | **2** | **3** | **4** | **5** |
|  |  |  |  |  |  |
| **How acceptable was the iREHAB programme to you?** | **Completely unacceptable** | **Unacceptable** | **No opinion** | **Acceptable** | **Completely acceptable** |
|  | **1** | **2** | **3** | **4** | **5** |
|  |  |  |  |  |  |

## Additional file 5: Modified TFAQ (standard care)

You have received this questionnaire as you received standard care as part of the iREHAB study. Standard care is the usual care that is currently given to patients who have been in intensive care. In many cases rehabilitation is not provided as part of standard NHS care when people go home from hospital after an admission to intensive care. This is standard care in the NHS.

We are seeking to gain additional information about your opinion of this standard care. There are 8 questions which will take you approximately 5 minutes to complete. For each question, select the number on the 5-point scale that most accurately reflects your opinion. There are no right or wrong answers. Please ensure to submit the form once completed.

| **Did you like or dislike the standard care you received?** | **Strongly dislike** | **Dislike** | **No opinion** | **Like** | **Strongly like** |
| --- | --- | --- | --- | --- | --- |
|  | **1** | **2** | **3** | **4** | **5** |
|  |  |  |  |  |  |
| **How much effort did it take you to participate in the standard care that you received?** | **No effort at all** | **A little effort** | **No opinion** | **A lot of effort** | **Huge effort** |
|  | **1** | **2** | **3** | **4** | **5** |
|  |  |  |  |  |  |
| **How fair is the standard care you received for people following and/or recovering from critical illness?** | **Very unfair** | **Unfair** | **No opinion** | **Fair** | **Very Fair** |
|  | **1** | **2** | **3** | **4** | **5** |
|  |  |  |  |  |  |
| **The standard care you received has improved your quality of life.** | **Strongly disagree** | **Disagree** | **No opinion** | **Agree** | **Strongly agree** |
|  | **1** | **2** | **3** | **4** | **5** |
|  |  |  |  |  |  |
| **It is clear to me how the standard care I received will help improve my quality of life** | **Strongly disagree** | **Disagree** | **No opinion** | **Agree** | **Strongly agree** |
|  | **1** | **2** | **3** | **4** | **5** |
|  |  |  |  |  |  |
| **How confident did you feel about participating in the standard care you received?** | **Very unconfident** | **Unconfident** | **No opinion** | **Confident** | **Very confident** |
|  | **1** | **2** | **3** | **4** | **5** |
|  |  |  |  |  |  |
| **The standard care I received interfered with my other priorities.** | **Strongly disagree** | **Disagree** | **No opinion** | **Agree** | **Strongly agree** |
|  | **1** | **2** | **3** | **4** | **5** |
|  |  |  |  |  |  |
| **How acceptable was the standard care you received to you?** | **Completely unacceptable** | **Unacceptable** | **No opinion** | **Acceptable** | **Completely acceptable** |
|  | **1** | **2** | **3** | **4** | **5** |
|  |  |  |  |  |  |

## Additional file 6: Modified TFAQ (staff)

You have received this questionnaire as you delivered the iREHAB programme to patients as part of the iREHAB study. We are seeking to gain additional information about your opinion of the iREHAB programme. There are 8 questions which will take you approximately 5 minutes to complete. For each question, select the number on the 5-point scale that most accurately reflects your opinion. There are no right or wrong answers. Please ensure to submit the form once completed.

| **Did you like or dislike the iREHAB programme?** | **Strongly dislike** | **Dislike** | **No opinion** | **Like** | **Strongly like** |
| --- | --- | --- | --- | --- | --- |
|  | **1** | **2** | **3** | **4** | **5** |
|  |  |  |  |  |  |
| **How much effort did it take for you to deliver the iREHAB programme?** | **No effort at all** | **A little effort** | **No opinion** | **A lot of effort** | **Huge effort** |
|  | **1** | **2** | **3** | **4** | **5** |
|  |  |  |  |  |  |
| **How fair is the iREHAB programme for people following and/or recovering from critical illness?** | **Very unfair** | **Unfair** | **No opinion** | **Fair** | **Very Fair** |
|  | **1** | **2** | **3** | **4** | **5** |
|  |  |  |  |  |  |
| **The iREHAB programme has improved patients' quality of life.** | **Strongly disagree** | **Disagree** | **No opinion** | **Agree** | **Strongly agree** |
|  | **1** | **2** | **3** | **4** | **5** |
|  |  |  |  |  |  |
| **It is clear to me how the iREHAB programme will improve patients’ quality of life.** | **Strongly disagree** | **Disagree** | **No opinion** | **Agree** | **Strongly agree** |
|  | **1** | **2** | **3** | **4** | **5** |
|  |  |  |  |  |  |
| **How confident did you feel about delivering the iREHAB programme?** | **Very unconfident** | **Unconfident** | **No opinion** | **Confident** | **Very confident** |
|  | **1** | **2** | **3** | **4** | **5** |
|  |  |  |  |  |  |
| **The iREHAB programme interfered with my other priorities.** | **Strongly disagree** | **Disagree** | **No opinion** | **Agree** | **Strongly agree** |
|  | **1** | **2** | **3** | **4** | **5** |
|  |  |  |  |  |  |
| **How acceptable was delivering the iREHAB programme to you?** | **Completely unacceptable** | **Unacceptable** | **No opinion** | **Acceptable** | **Completely acceptable** |
|  | **1** | **2** | **3** | **4** | **5** |
|  |  |  |  |  |  |

## Additional file 7: Analytical procedures

The analysis followed the Framework Method (Gale et al., 2013), progressing through a structured and iterative process to systematically code, categorise, and compare data from remote rehabilitation participants, standard care participants, and staff involved in care delivery.

**Stage 1: Transcription**

Verbatim transcription of interview recordings was undertaken by an external provider. All transcripts were checked for accuracy against the original audio files and de-identified to maintain confidentiality.

**Stage 2: Familiarisation with the data**

To ensure close engagement with the data, the researchers (CG and DM) read and re-read transcripts and revisited audio recordings to develop a detailed understanding of participants’ experiences prior to formal coding. Early reflections and potential sensitising concepts were discussed with BC and JB to support reflexivity and inform the analytic approach.

**Stage 3: Coding process**

Following familiarisation, CG and DM independently conducted line-by-line coding of an initial subset of eight transcripts, selected to represent variation in participant type (remote rehabilitation, standard care, staff). Descriptive codes were assigned to capture meaningful concepts or features within the data.

After independent coding, CG and DM compared and refined their coding lists through discussion, merging overlapping codes, removing redundancies, and clarifying ambiguous labels. The preliminary list was reviewed with BC and JB to ensure conceptual clarity and coverage of relevant data. This process resulted in an agreed initial set of codes that informed development of the working analytical framework.

**Stage 4: Developing the working analytical framework**

Codes from the initial eight transcripts were organised into a working analytical framework (WAF) encompassing all participant groups. The Theoretical Framework of Acceptability (TFA) constructs were used as overarching categories, with inductive codes systematically mapped beneath them.

Codes that did not align directly with the TFA were grouped into additional, thematically coherent categories to capture important contextual or emergent material. Each code was assigned a unique identifier to facilitate retrieval and ensure transparency.

The draft WAF was reviewed collaboratively with BC and JB to check conceptual coherence, ensure alignment with TFA constructs, and confirm that inductive elements were retained. Revisions were made through iterative discussion until consensus was achieved on the final structure.

**Stage 5: Applying the analytical framework**

The final WAF was systematically applied to all transcripts. CG and DM each coded approximately 50% of the dataset, with 20% double-coded to assess consistency and ensure shared interpretation. Discrepancies were resolved through discussion, and periodic team checks with BC and JB ensured continued alignment with the agreed framework and theoretical underpinnings.

**Stage 6: Charting the data into the framework matrix**

Coded data were summarised into a framework matrix generated in NVivo and exported to Excel for further analysis. The matrix arranged participants as rows and codes as columns, allowing comparison both within and across cases. Charting involved abstracting key points while retaining sufficient contextual detail for interpretive depth. References to illustrate “good quotes” were embedded in the matrix to support traceability. The matrices were reviewed by BC and JB for completeness and fidelity to source data.

**Stage 7: Interpreting the data**

Interpretation focused on identifying patterns of convergence and divergence across patient and staff perspectives. The analysis considered how acceptability constructs interacted and how these dynamics varied by recovery context (remote rehabilitation versus standard care).

Theme development involved grouping related codes into higher-order categories, reflecting both shared and contrasting experiences. CG and DM led this interpretive process, with regular team meetings involving BC and JB to review and refine emerging themes.

Final themes were agreed collaboratively and supported by illustrative quotations labelled by participant type (RR = Remote Rehabilitation; SC = Standard Care; Staff). This process maintained theoretical consistency while enabling nuanced, cross-group comparison of how acceptability was understood and experienced.

## Additional file 8: Coding tree

| **Theme / TFA construct** | **Analytical interpretation** | **Convergence and divergence across groups** | **Illustrative quotes** |
| --- | --- | --- | --- |
| **Early recovery -** A shared sense of falling through the gaps | Early recovery shaped perceptions of acceptability. Accounts revealed a disjointed transition from hospital to home, where the absence of guidance and reassurance left survivors feeling unsafe and unprepared. Continuity and reassurance were seen as vital in the immediate post-ICU phase. | Patients experienced the lack of follow-up as personal abandonment and emotional neglect, while staff framed it as a systemic failure due to limited integration and resources. | *“I fell through a hole in the NHS and social care, I was left alone”* (RR11)  “*They gave me nothing, just sent me home with a box full of pills”* (SC03).  *“for most people, they do just say that the care in hospital was really good […] but as soon as they left hospital, that really just dropped off. A lot of them had minimal input or no input”* (Staff02).  “*I had no advice, no instructions. Nobody came to see me, nobody came near me, […] I really felt abandoned*” (RR14).  “*Participants just don’t know where to go for the information*” (Staff03).  “A*bsolutely terrifying, in all honesty. Overwhelming. New fears unlocked*” (RR29).  “*literally they’re crawling on their hands and knees going up the stairs.*” (Staff01). |
| **Affective attitude -** Connection as foundational to recovery | Connection underpinned positive affective attitude and, in turn, acceptability. Regular, personalised contact through remote rehabilitation fostered reassurance and motivation, whereas its absence led to uncertainty and disengagement. | Remote participants valued relational continuity and personalised care; standard care participants highlighted prolonged isolation and lack of feedback; staff emphasised the need to humanise contact as essential to acceptability. | *“Because somebody is there, telling you, ‘Come on, we’re here to help […] whatever you’re going through, we can help you.’ It takes away the apathy involved”* (RR15).  “*I think just making it more humanised is really important for people*” (Staff03).  *“the bit that got to me most of all, is just being left alone for months and months at a time with no feedback”* (SC03).  “*You’re in your own home […] where you feel safe*” (RR11).  *“All I wanted to do was get out, get out and get home. And once I got out and got home, I didn’t realise how hard it was going to be, you know. It was. It was tough.”* (SC30).  “*Without you ringing, I would’ve been totally isolated*” (SC01)  *“She could see my level… and where I was getting to”* (RR02)  *“I just want to push my grandchild up the road… I’m not going to struggle to do aerobics”* (SC01).  *“You could have the most perfect manual on the planet, but if you don’t engage with the person, it probably won’t be completed”* (Staff07). |
| **Burden** *-* Effort that counts versus effort that costs | Acceptable burden reflected not low effort, but effort that held meaning and recognition. When effort was structured and shared with professionals, it was experienced as worthwhile; when unsupported, or a burden to families, it felt depleting and costly. | Remote rehabilitation participants framed effort as motivating investment; standard care participants described relentless exhaustion and reliance on families; staff perceived burden more acutely, seeking to protect participants from overload. | *“The more you put in, the more you will get out of it”* (RR02).    “*The physical exercise was a slight shock to the system that left me feeling tired, but I had achieved something, so it was quite good*.” (RR16).  *“We forget that, if we ask them to invest in the whole intervention, that is three appointments a week… It could be quite a lot for a participant to take on.”* (Staff S03)  *“If I’m going anywhere, I have to think about how I’m going to get to the place […] I have to try and plan it and find out where I’ve got to go”* (SC01).  *“My wife as well, how she got through that first month with me, I don’t know. She should have been able to talk to somebody”* (SC30).  *“Some of them will say, ‘It wasn’t me, it was my partner that took time off work and ran the house in the background’*” (Staff04).  *“I think it does naturally just affect you a bit but nothing ever too extreme”* (Staff02). |
| **Opportunity costs -** Balancing recovery with everyday responsibilities | Opportunity costs were acceptable when rehabilitation integrated smoothly into daily life and aligned with participants’ values.  When flexible and clearly beneficial, investment was justified; when unstructured or disruptive, recovery was deprioritised. | Remote participants viewed rehabilitation as time well invested; standard care participants prioritised daily routines and energy conservation; staff saw flexibility and adaptability as central to making opportunity costs acceptable. | *“So you just put aside 30 minutes because it’s your health, isn’t it? So if you can’t devote 30 minutes to helping to improve your health, then you know*” (RR31).  *“Because of that flexibility that was built in, it didn’t actually disturb anything else”* (RR33).  *“It’s fitting it alongside their recovery, rather than stopping what they’re doing to allow it”* (Staff04).  *“Well, I probably do a couple of miles […] The longer walks are occasional, and […] I will not go out in the rain unless I have to*” (SC17). |
| **Ethicality -** A right not a privilege | Moral acceptability rested on perceptions of fairness and inclusion. When access to rehabilitation felt equitable, support aligned with participants’ moral expectations of post-ICU care; when it appeared selective or exclusionary, it undermined trust in the system’s legitimacy. | Remote participants expressed gratitude tinged with unease about others’ exclusion. Standard care participants interpreted exclusion as neglect of their needs. Staff viewed inequitable provision as an ethical failure of the system, highlighting digital and structural barriers that limited reach. | “*Like you say, not everyone got the opportunity to try*” (RR09).  “*I mean I’m one of the lucky ones, aren’t I, who have had it… perhaps it’s not so fair*” (RR09).  “*It should become more standardised across for everybody to be able to access*” (Staff03).  *“I think they deal with drug people and insulin on a daily basis. So I imagine those are the priorities”* (SC04).  “*I wish I’d heard something from the group I’d been put into […] maybe they think that because of the questionnaires, it didn’t look like I had a problem*” (SC01).    *“I’m normally fairly technically literate so being online was no problem*” (RR 16)  “*Even with the older population, they’re very wary about downloading or installing other applications*” (Staff01). |
| **Coherence -** Making sense of recovery | Acceptability was enhanced when rehabilitation helped participants make sense of recovery as a connected biopsychosocial process. Integrating these components created coherence; without such integration, recovery felt fragmented and confusing. | Remote participants developed coherence through structured support and professional contact; standard care participants sought coherence through comparison or shared experience but often struggled to achieve it; staff described coherence as actively built through communication and goal-setting. | “*It gave you a way of seeing a future […] that you were going to get a little bit better, even if it might take a while*” (RR34).  “*Part of our role is reminding them how far they’ve come since week one*” (Staff03).  *“the perfect tool […] to holistically look at the body, mind, and soul*” (RR11).  “*I don’t want to overdo it and undo all the good*” (SC25)  “*I suppose they rank your heart with your brain as critical areas of your body to be just totally ignored*” (SC03).  *“My favourite was the peer group because it made you realise you’re not the only one.”* (RR11  *“sometimes it’s really difficult to hear other people talk about their struggles, and also to have to talk about your struggles. It can be upsetting and traumatic.”* (RR12).  *“I just think sometimes just to talk to somebody who’s been through it or going through it, it’s that old saying, a problem shared is a problem halved”* (SC20). |
| **Perceived effectiveness -** Seeing, feeling, and questioning progress | Acceptability was shaped by whether rehabilitation was perceived to “work.” Tangible progress legitimised effort and strengthened belief in recovery; in its absence, doubt and loss of motivation were common. | Remote participants described visible gains and regained agency; standard care participants expressed doubt and fragility; staff viewed progress and continued self-management as key markers of effectiveness. | *“When I first had my sit and stand test, I couldn’t do one, when I had my second one I did seven or eight so that’s an amazing change*” (RR11).  “I*t gives you your life back mentally, emotionally, and physically. It’s the best thing I’ve done” (*RR11).  “*They’re doing more than what they were doing before the study […] it’s still going to be really beneficial in getting them back to where they were at*” (Staff02).  *“I’m driving again. I wasn’t allowed to drive for up to three months. I now drive again”* (SC03)  *“I would like to get back to some normality, but I know I’m nowhere near going back properly yet. I’m just not physically strong enough yet”* (SC19).    *“I get very depressed and I don’t understand why I get depressed, but I saw my doctor yesterday and he just said, “Because your mind hasn’t come to terms with this major shock you’ve gone through”* (SC03). |
| **Self-efficacy -** Gaining and losing trust in one’s own recovery | Acceptability depended on participants’ confidence to re-engage with their bodies and recovery. Structured rehabilitation enhanced self-efficacy through guided practice and reassurance, transforming fear into action. Without such support, uncertainty and self-doubt restricted participation and reduced acceptability. | Remote participants described growing confidence through guided mastery; standard care participants reported persistent fear and self-doubt; staff viewed graded encouragement and skill-building as essential to rebuilding confidence. | “*It was the best bit […] knowing what to do, […] and how to do it safely. Because that’s why I wasn’t feeling confident, before iRehab, to exercise, because I didn’t know what to do that wouldn’t hurt me […] I didn’t know how to exercise safely. And then I learned how to do it, and I became much better, much quicker.” (*RR12).  *“I think I would’ve found it very difficult without the support and encouragement […] Having the encouragement helped me to get going”* (RR14).  *“Patients sometimes need that guidance […] ‘You might not have the balance, but we can do this seated… You can take it a bit slower’”* (Staff01).  *“Earlier on, that would’ve helped, to talk to somebody, to see what I was capable of doing. Because a lot of it is you’re scared to push yourself, in the beginning, to see actually how much you can do”* (SC01).  *“I can remember waiting and waiting for somebody to come. And I just thought, I’m gonna just do this by myself”* (SC35).  *“I’m still suffering from a lack of confidence in silly things. I get worried a lot when I never used to worry”* (SC03).  *“I think when you come out of ICU, patients lose trust in their own ability to make those decisions. And I think giving them that space to start gaining their own trust back, that they can maybe walk to the bottom of the garden and back, or they can, you know, that they can get that confidence in what they can do back themselves.”* (Staff04). |

## Additional file 9: Overview of themes, subthemes and quotations

| **Construct** | **Themes** | **Arm notes (RR/SC/Staff)** | **Quotes/words** |
| --- | --- | --- | --- |
| **Initial recovery context:** The broader needs, challenges, and circumstances that make rehabilitation care relevant or necessary, rather than how participants perceive care itself | **Being discharged as a cliff edge** - Acceptability of recovery was undermined when discharge felt abrupt and unprepared. The sharp withdrawal of professional support created fear and uncertainty, leaving families to take on responsibilities they felt ill-equipped for. | RR: Acute care praised, then a sharp drop in contact; iRehab later fills the gap.  SC: Repeated sense of nothing after discharge, equipment not delivered, no physio.  Staff: Acknowledge limited post-ICU provision, bed pressures shaping discharge. | “I fell through a hole in the NHS and social care, I was left alone.” (RR11)  “There’s nothing. When you get discharged, you get nothing.” (RR12)  “I literally wasn’t offered a single bit of physio, which I was quite shocked about.” (SC20)  “Inside the hospital, they’re absolutely incredible. And some of the kindest people I’ve ever met. But when you’re out of their care, it is a bit kind of like you’re thrown in a big pond, and you’re just sat there waiting for the rod to come in.” (RR29)  “I think really, for most people, they do just say that the care in hospital was really good and there was lots of staff there. They could always have people to support them but as soon as they left hospital, that really just dropped off. A lot of them had minimal input or no input (Staff02)  So once they’ve done the operation, there is no follow up. There’s nothing else to do. They fixed it. (SC04)  “I think the provision for patients, after ICU, is very limited. Even when you look, generally, across the NHS, as soon as they hit the ward, it’s normally a completely different team” (Staff03) |
|  | **All-encompassing disruption** - The scale of physical, cognitive, and psychological decline made early recovery feel overwhelming. Acceptability depended on whether adequate scaffolding was in place to support even basic tasks. | RR: Returned home profoundly weak (wheelchair/rollator, sleeping downstairs, commode). Basic transfers were exhausting; some had tracheostomy-related speech issues and major weight loss. iRehab later provided structure.  SC: Reported no physiotherapy and had to self-manage (Zimmer frame, tins/TheraBands, strip washes). Open wounds and pain limited early activity; equipment and community services often delayed.  Staff: Describe extremes of debility at home, including people crawling on hands and knees up stairs; recognise minimal post-ICU rehab capacity. | “I got home and I was just exhausted.” (RR02)  “Absolutely terrifying, in all honesty. Overwhelming. New fears unlocked.” (RR29)  “I’ve had one particular participant, or even a few actually who’ve said that literally they’re crawling on their hands and knees going up the stairs.” (Staff01)  “It was like I was climbing up Everest” (SC20)  You come out of ICU, and you don’t know where you are, you don’t know what’s real… you know? (SC01) |
|  | **Uncertainty and confusion** - Poor orientation and lack of timely information made recovery feel unsafe and unacceptable. Participants questioned their ability to cope when left to self-manage, contrasting with the reassurance when guidance was clear and immediate. | RR: Reported minimal guidance at discharge; follow-up calls or GP access were delayed or unhelpful, leading some to give up seeking support; iRehab later filled the informational void.  SC: Expected condition-specific guidance but received none; described discharge as transactional (“box of pills”) and had to self-initiate referrals; confusion around tests (e.g., sit-to-stand instructions).  Staff: Acknowledge post-ICU provision is limited; note that participants often do not know where to go for information. | “They gave me nothing, just sent me home with a box full of pills.” (SC03)  “I was discharged with nothing more than a list of medication, that’s all. I had no advice, no instructions. Nobody came to see me, nobody came near me, it was… I really felt abandoned.” (RR14)  “I think, sometimes, it’s just about… Participants just don’t know where to go for the information.” (Staff03) |
| **Affective attitude:** What participants liked, disliked, or found meaningful in their experience of iRehab or standard care | **Supportive contact and reassurance -** Rehabilitation was most acceptable when people felt supported and reassured. Human contact provided validation and reduced isolation, while absence of follow-up created feelings of abandonment. Variation in preferences, such as avoiding peer groups, reflected personal differences rather than barriers to acceptability. | RR: One-to-ones felt like a safe space; reassurance about symptoms and paced progression increased confidence and sense of feeling ‘seen’  SC: Anger and sadness at being left without follow-up; relief when even minimal empathy or contact was offered, even brief trial contact felt emotionally protective. Some self-identify as “private,” would avoid peer support groups but others found ‘ICU steps’ as beneficial for shared connections  Staff: Emphasise normalising emotions; see clear benefit in simply “having someone to talk to” beyond family. | Without you ringing, I would’ve been totally isolated” (SC01)  “It means that you care and nobody else does.” (SC30)  “I mean, I came out, I’ve been in ICU, I’ve been in a coma and everything and after three months, nobody ever contacted me to see, to say, are you OK? You know nothing. Nothing at all, which I think bad.” (SC30)  “Communication, better communication, not just being left for weeks and weeks not knowing… That was the bit that got to me most of all, is just being left alone for months and months at a time with no feedback.” (SC03)  “Nobody at all. The family. But nobody like… she could have just done with a little phone number saying if you’re in trouble, give us a ring. What a difference that would have made.” (SC30)  “If I was having a particularly down day, [the interventionist] could pick me up in no time so it worked very well.” (RR14)  “Because somebody is there, telling you, “Come on, we’re here to help. You’re not on your own. Loads of people are going through this. We’re part of an organisation. There’s a whole thing behind this. So whatever you’re going through, we can help you.” Yeah? It takes away the apathy involved.” (RR15)  “One to one, talking with [the interventionist], she’s a health professional, it’s her job, and after the first or second session, it was talking to a friend.” (RR16)   “And it’s having the space to talk to somebody who just feels like your person to talk to. Do you know what I mean? They’re there because they have a purpose and they understand “ (Int34)  “People would feel that at least something was being done.” (SC08)  “It’s also kind of that rapport just gives you, not credit, but like the patient think like I’m being listened to you.” (Staff01, t2)  “A lot of them are older so it’s maybe just their wife or their husband who’s been through it all, so it’s already been quite a big emotional burden on them, so it’s nice to have somebody else they can talk to to kind of take that weight off.” (Staff02)  So, yeah, I think it’s just reiterating that it’s normal, that a lot of the things that patients are experiencing are normal. It’s okay to also be upset, sometimes (Staff01)   I think just making it more humanised is really important for people (Staff03) |
|  | **Value of personalised care** - Acceptability depended on rehabilitation feeling tailored to individual needs, goals, and capacities. Personalised adaptations sustained trust and motivation, while generic or irrelevant approaches risked disengagement. | RR: Strong appreciation of real-time adaptations (e.g., pinned shoulder alternatives; adjusting reps/pace).  SC: SC participants emphasised the absence of personalised input, often expressing frustration at generic or irrelevant advice.  Staff: Tailoring is fundamental; one template won’t fit everyone. | “I mean, I’m supposed to have had a form from the nurses to say, and take pictures of my wound. Well, I never had the form.”  “Because she could see my level, what I was [ ___ 0:50:33] and where I was getting to.” (RR02)  “I mean, [the interventionist] advised me not to use the dumbbell, you know, the things you lift? She said, “You want to take care there”, because I mean I could sort of strain myself or pull my bones or something like that.” (RR07)  “I don’t want to do aerobics. I just want to push my grandchild up the road in a pushchair. Yeah, I don’t like- I’m not going to struggle to do aerobics. I’ll struggle to look after my children and my grandchildren.” (SC01)  The only thing I would say is that, sometimes when we were discussing whatever, what was in front of me in the book was being read out to me. Fair enough. It just seemed superfluous. I could read it to myself and had probably read it beforehand anyway, so that wasn’t advancing anything. She was just reiterating what was in front of me. Other than that, no, everything seemed to be very good. (RR16)   “So you could have the most perfect manual on the planet, but if you don’t engage with the person, it probably won’t be completed” (Staff07) |
|  | **Navigating recovery at home** - The home environment enhanced acceptability by offering privacy, safety, and convenience. However, when people were left to self-manage without structured scaffolding, home could also become a source of anxiety and uncertainty. | RR: Consistently valued home-based delivery for comfort, safety, and flexibility; some exercised in the garden, used furniture for support, and highlighted reduced travel/anxiety.  SC: Often valued the comfort of being at home but described feeling abandoned when no structured support was available. Even hypothetical remote or video contact was viewed as valuable when no structured support existed. One participant had experienced remote care previously so recognised it’s value  Staff: Saw home as an acceptable and realistic site for rehabilitation delivery but emphasised the need for ongoing support. | That’s another thing, it was nice to do it in your own home over this, you’re not sitting in a room where you’re like, “I’m only here because I’m desperate and I’m not bringing my oxygen cylinder with me.” (RR02)  “It makes it easier, for you, that you’re in your own home and you’re able to turn on Microsoft Teams to see the exercises and the peer group, because you’re in a comfortable place, to start with, where you feel safe. Doing it online, you can see what you’re meant to do and it’s perfect.” (RR11)  “Oh, yes. The exercise sessions as well, it was perfect. Because then you’re sitting in your own house and you can go and have a shower.” (RR02)  “Within the confines of the house, obviously… you know where everything is. I was able to hold onto chairs, onto the worktops and things like that.” (RR07)  “It makes it easier… you’re in your own home and you’re able to [use] Microsoft Teams… because you’re in a comfortable place, to start with, where you feel safe.” (RR33)  “I actually preferred the remote… it was just time-saving and more comfortable, because I could choose the locations myself. A couple of times, because the weather was good, I did the exercises in the garden.” (RR33)  “You would’ve thought my name would’ve been somewhere on their system, for them to actually ring me and ask if I needed anything to help me live at home. You know?” (SC01)  “I think so. I think people would feel that at least something was being done. Even if it’s remote sessions or group video sessions, I think people would appreciate that.” – (SC08)  “The one thing I did have the first time was the physiotherapy, but because it was COVID times it was all on Zoom. I found that so amazing, it got me so much fitter and so much stronger.” (SC34)  But all I wanted to do was get out, get out and get home. And once I got out and got home, I didn’t realise how hard it was going to be, you know. It was. It was tough. (SC30)  “It means people don’t have to travel and spend time travelling” (SC01) |
| **Burden** | **Managing the weight of recovery -** Rehabilitation required sustained physical and emotional effort. Acceptability depended on whether this effort felt purposeful and supported. Without visible progress, reassurance, or safe spaces, the demands of recovery risked becoming overwhelming or wasted. | RR: Effort reframed positively when tied to progress, reassurance, or validation.  SC: Burden stemmed from self-managing without structure, leaving effort uncertain, unsupported, and added to by logistical challenges.  Staff: Framed strain as inevitable but acceptable if paced and normalised. Also reflected on own strain, but deemed worthwhile | “The physical exercise was a slight shock to the system that left me feeling tired at the end of it, but I had achieved something so it was quite good.” (RR16)  I mean it obviously required a bit of effort on my part, but I didn’t feel bad about it or nothing like that. I mean, it’s went, and as the weeks went by, it’s got easier, you know? (Int07)  “We forget that, if we ask them to invest in the whole intervention, that is three appointments a week… It could be quite a lot for a participant to take on.” (Staff S03)   “The more you put in, the more you will get out of it.” (RR02)  I mean, admittedly, going through some of the exercises I’m like, “God, I hate this.” but that’s exercise, it’s normal. It’s been 100% perfect for me, I wouldn’t have changed anything on it. ( RR11)  “Then the exercise made me feel alive again… I was always tired because I pushed myself too far. I always had to sit for an hour afterwards and have a couple of cups of tea.” (RR11)  it’s a bit frustrating that I’m the one having to chase this. (SC08)  But if I’m going anywhere, I have to think about how I’m going to get to the place. Like going to the hospital, it’ll be, “How can I get there? I can park in the disabled place, but how am I going to walk from the disabled place to where I need to go?” I have to try and plan it and find out where I’ve got to go. (SC01)  They don’t make you upset but kind of like just… You kind of feel a bit… You feel empathetic. I don’t know if empathetic or compassionate, or which one it is, but you do feel for them. Actually, their joy is also your joy. Actually sometimes, also, their… I think that’s especially as you build up rapport with patients. Some patients, it’s a bit easier to do that than others. I think any challenges that they have… I think it’s only human to feel a wee bit of that too. (Staff01)  “You’re talking to people and they’re getting upset, they’re getting emotional, on the screen. I think it does naturally just affect you a bit but nothing ever too extreme or anything. Yeah, I suppose it’s just hearing other people’s stories and just how they have been affected by it.” (Staff02) |
|  | **Family as hidden carriers of burden -** Acceptability was influenced by how responsibility for recovery shifted onto families. When support for carers was lacking, this reliance felt unfair and unsustainable, undermining the feasibility of rehabilitation for both patients and relatives. | Int: Family members supported intimate care (washing, toileting, showering) but some of burden aleviated by support from irehab  SC: Family took on care coordination, and emotional strain; role reversals noted.  Staff: Reflected on hidden family load and argued future iRehab should include family support. | “My mum was coming in and bathing me on the toilet and doing the whole bed bath with me on the loo.” (RR02)  “Mum was still helping me wash.” (RR02)  They’re trying to support you and you think how many times do they want to hear this? They don’t. But until you can work it through yourself in your own mind, you do need that support. (RR34)  “Well, like cooking meals. My husband had to be up with me during the night, if I needed to go to the toilet. I had the commode, but I couldn’t actually stand up on my own, so he had to help me to sit on the commode. Yeah, it was really difficult.” (SC01)  “The family support is as important as me, believe me.” (SC30)  “It was horrible for my poor husband… Although he wasn’t working full time and he was doing most of his work from home, it was disrupting him as well.” (SC20)  “But I think if you could, especially in the first month… my wife as well, how she got through that first month with me, I don’t know. She should have been able to talk to somebody.” (SC30)  “Well, she did all the cooking. For three months she did all the cooking, because I cook in the house because I love cooking. I was getting breakfast in bed, meals in bed… I was being pampered.” (SC03)  “Some of them will say, ‘It wasn’t me, it was my partner that took time off work and ran the house in the background.’” (Staff04)  “I think with iRehab, it probably needs to have another branch or strand to it...which is family. (Staff04) |
| Ethicality | **Perceptions of fairness in access -** Acceptability was filtered through perceptions of fairness. Random allocation or threshold-based access felt ethically troubling, while universal provision was viewed as the only equitable way to deliver rehabilitation. | Int: Described feeling “lucky” to receive iRehab and aware that others had to cope without equivalent support.  SC: Spoke of being excluded or “not bad enough,” with frustration at being denied the rehabilitation support.  Staff: Highlighted the importance of making rehabilitation universally available, not reliant on chance | “Like you say, not everyone got the opportunity to try” (RR09)  “Everybody should get a chance” (RR09)  “I think they deal with drug people and insulin on a daily basis. So I imagine those are the priorities. (SC04)  “No, I don’t think so. I probably would’ve been better off being chosen for the other group, and then I could’ve met other people that have been through the same thing, and yeah, see where I went with the exercise.” (SC0)1  “I mean I’m one of the lucky ones, aren’t I, who have had it but, somebody who hasn’t had it, perhaps it’s not so fair. ( RR09)  I guess it was a little bit disappointing, because it would have been nice to find out what extra support was given… if I was in that group, I’d probably get more support… it would have been nice getting there, but I completely understand… some people have to get the regular one and some people get the extended one.” (SC23)  “What you guys did is what the NHS should have done. In my opinion. Like I said, it was six weeks plus until I’d heard anything from them. And if it hadn’t have been for iRehab, I would have done all that on my own, which is quite daunting.” (RR29)   I wish I’d heard something from the group I’d been put into. I don’t know what the criteria is for that. I mean, maybe they think that, because I- you know, filling in the questionnaires, it didn’t look like I had a problem. (SC01)  I think that probably just makes me think, “This is why we need to do what we’re doing.” Hopefully, if this trial does show success as well, then more things like that will be made more accessible and maybe we won’t have to rely on charities and things like that. It should become more standardised across for everybody to be able to access. (S03) |
|  | **Technology as gateway and gatekeeper -** Remote delivery was acceptable when digital access and support were in place, but inequities emerged where technology, literacy, or resources limited engagement. This raised ethical concerns about exclusion while also creating practical burdens, with staff investing time troubleshooting and participants sometimes reverting to phone delivery. To ensure fair access, technology needed to be as simple and streamlined as possible. | RR: Mixed experiences - majority found video conferencing straightforward, others struggled more and reverted to telephone  SC: N/A as no tech required due to not engaging in the intervention  Staff: Viewed video as essential for rapport and safe demonstration, but noted that troubleshooting and digital confidence varied widely. | “Yeah, I’m confident using stuff online. So for myself, that would be perfect really. But other people, maybe not so much (…) I know some people might prefer one-to-one, but for me, remote would be perfect.” (SC23)  “Oh, I was quite happy with it, because I couldn’t do it on Zoom, because I’m a bit of a technophobe (…) But we did it over the phone, and that’s no problem.” (RR07)  “Yeah, initially I was like, ‘Oh, you know, is this going to work for me?’(...) I’m not a good IT person but it was very straightforward. It came in on the email and, yeah, I just clicked on it so it was very simple.” (RR09)  “I’m normally fairly technically literate so being online was no problem. Having said that, we did have some bad connections. The picture sometimes froze or the sound glitched, but that’s part and parcel of the net.” (RR16)  “Demonstrating bends and stretches over the telephone is a bit tricky.” (RR31)  “So I think that’s the only drawback I could envisage… is if someone’s very much older or has been away from technology for a long time.” (RR34)  “One of the difficulties would definitely be… the technological difficulties about having two systems, Microsoft Teams for their one-to-ones and then Beam plus Zoom for their other group elements. It proves to be quite challenging.” (Staff01)  “Even with the older population, they’re very wary about downloading or installing other applications. That’s another one that they have to, or maybe another two that they have to, sign up to, that they’re not really too sure or comfortable doing.” (Staff01)  “Some people… you’ll try for weeks and weeks, and in the end you just revert to a telephone appointment, because it’s quite hard to get everything done within the hour, especially if you spend the first 15 minutes troubleshooting tech issues.” (Staff 01 T2)  I also think transferring to beam was a big thing, a huge thing. It was a pain to say it lightly, I don’t even think just a pain for myself or a pain for clinicians, but also a pain for participants. (Staff01_t2)  “These people are like…. they’re not very willing to give even one software ago or one interface to go. And you’re trying to ask them, OK, can we do over two. And it was just mangled I think.” (Staff01) |
| **Coherence** | **Sense-making through shared experiences -** Peer contact supported coherence by normalising symptoms and reducing isolation. When effective, these shared exchanges made recovery feel more comprehensible and less overwhelming, though the value placed on peer support varied across individuals. | SC: Often described peer contact as the only source of support hence they placed high value on validation and reassurance from others in similar situations.  RR: Some participants appreciated the peer groups for normalisation, but tended to rely more on one-to-one professional input, so peer groups were seen as supplementary and sometimes less essential or uncomfortable  Staff: Saw peer support as beneficial for reinforcing understanding and reducing isolation, but recognised it was not suited to all participants. | “I just think sometimes just to talk to somebody who’s been through it or going it, it’s that old saying, a problem shared is a problem halved.” (SC 20)  “I think that’s it is going and hearing from other people that you’re not alone in this and that there is one, it’s normal. And two, there is a future” (SC 30)  “The fact that we had the peer review group and we were talking to people who had gone through similar, although not the same, makes you feel part of a group and that you’re not alone” (RR11)  “I just absolutely loved it, the exercise classes and everything, but my favourite was the peer group because it made you realise you’re not the only one who has had such a long time in ICU. That made me feel that I was in this very exclusive group, which was quite nice. In fact, I’ve kept in touch with two of the people since because they asked, in the group, if they could keep in touch with me.” (RR11)  “Sometimes it’s really difficult to hear other people talk about their struggles, and also to have to talk about your struggles to other people, so sometimes it can be, like, upsetting and traumatic.” (RR12)  “You know that if you explain to them in detail what happens […] then you might convince them towards going into the peer support group. But then there’s the other category which are just...you know, from the moment you ask them in the first appointment, it’s a hard no.” (Staff07) |
|  | **Recognising recovery as an ongoing process -** Rehabilitation was more acceptable when framed as gradual and progressive. Clear guidance, milestones, and goal-setting sustained motivation, whereas uncertainty about what was safe or “allowed” fuelled anxiety and fear of harm. | RR: Participants valued setting goals and tracking progression with exercises. This helped them monitor improvement and gave recovery a sense of direction.  SC: Participants relied on personal milestones and symptom reduction as markers of progress but often felt anxious about doing the wrong thing without guidance.  Staff: Saw their role as reminding participants of progress already made, reinforcing confidence and framing recovery as stepwise. | “I don’t want to overdo it and undo all the good, but I’m accepting now that yes, it will… It might take me the full six months. I wasn’t expecting it because when they first said six-month recovery, I just scoffed at it and I thought, no, that’s not me. But it actually is and it’s learning to accept now that yes, it might.” (SC25)  “Just a bit of advice on how far I can push it would have been helpful.” (SC25)  “I was thinking if I did too much exercise it would’ve split the stiches or damaged the wound, so that was always at the back of my mind.” (SC19)  “I feel I made a good progression for somebody of my age because… you think to yourself, is it your recovery or is it you’re older now and you shouldn’t be able to do things as fast? But I feel I’ve made a good recovery and a good progress.” (SC18)  “It gave you a way of seeing a future, if you know what I mean. That you were going to get a little bit better. Even if it might take a while.” (RR34)  “The recovery pack reiterates that (…) it’s common to feel fatigued, it’s common to have breathlessness after ICU… patients have said that just having that makes them feel, ‘I’m not alone’.” (Staff01)   “Actually it is really important that they do start doing that, so I think having somebody saying, “Okay, that is okay to do.” is quite helpful in their rehabilitation as well.” (Staff03)  “Part of our role is reminding them how far they’ve come since week one.” (Staff03) |
|  | **Fitting the pieces together -** Acceptability increased when physical, psychological, and educational elements were understood as interconnected. Narrow or fragmented delivery risked making rehabilitation feel less relevant and less acceptable. | SC: Rehab was often experienced as narrowly physical (e.g., walking or physiotherapy), but participants recognised that recovery was broader, involving psychological and informational support that was often missing.  RR: Many valued the multi-component nature of iRehab and how it linked physical progress with emotional recovery and practical knowledge.   Staff: Saw coherence as a strength of iRehab, where integrated components filled gaps in standard care. | “I suppose they rank your heart with your brain as critical areas of your body to be just totally ignored. (SC03)  “When I read the booklet, it explains a lot more what happens (…) I was genuinely shocked, I didn’t realise how severe. It made sense though because I thought that makes sense as to why I feel the way I do.” (SC20)  “Yes, I just think the whole package was good, I can’t fault one of the sessions(…) all of them, as part of the package, was why it worked, I think.” (RR02)  “I just think that the iRehab trial is the perfect tool to help those people who’ve had critical illness and help them adjust to life on the outside and to holistically look at the body, mind, and soul. So you’re looking at the mind, you’re looking at the physical aspects of the exercise, and you’re also looking at what they’re feeling inside. I think it covers everything it can affect.” (RR11)  “My GP didn’t want to know. At the time, when I came out, I asked for counselling, from the GP, and she said, “No, you don’t need it.” So that was knocked back.” (RR15)  “So I think it does need to be kind of participant-led and holistic, and look at a broader spectrum of things.” (Staff04)  You know, I think this is a real strength of this...that it is holistic, broad, and patient-menu-based, if you like, rather than, you know, rather than condition-specific where you only see that one team that deals with those problems. (Staff04)  “It’s a journey, and it’s an ongoing journey (…) not just purely physical. It’s the motivation to get up and walk, the pain, the weakness, the loss of confidence, the fear that something’s going to happen again.” (Staff04)  So when you see them in iRehab, it is a physical journey, but you also have that kind of, I suppose, cognitive and psychological aspect, which is really important for behavioural change. (S04) |
| **Opportunity costs** | **Making space for recovery** - Acceptability depended on whether rehabilitation competed with other life demands. For some, it was straightforward to accommodate or felt worthwhile to prioritise. Others found fixed timings of group sessions harder to balance with work, family, or recovery needs. Flexibility in scheduling reduced these opportunity costs, making participation more feasible. | SC: Rehab was rarely seen as interfering, though practical barriers such as weather could limit activity more than competing priorities.  RR: Most participants fitted sessions in with little difficulty, but group components often conflicted with work or family responsibilities. Flexibility and rescheduling were valued for reducing disruption.  Staff: Acknowledged that three components per week could compete with other appointments and emphasised the need for adaptable scheduling. | “So you just put aside 30 minutes because it’s your health, isn’t it? So if you can’t develop 30 minutes to helping to improve your health, then you know” (RR31)  “So it’s fitting it alongside their recovery, rather than stopping what they’re doing to allow it, you know.” (Staff 04)  “No, not at all. It did not interfere ‘cause you know if you’re going to get contacted just to leave that period free.” (SC27)  “I probably do a couple of miles, two or three miles every day, just simply, as I was today, walking down the local high street doing some shopping. The longer walks are occasional, and it’s weather. I will not go out in the rain unless I have tp.” (SC17)  “Sometimes I was struggling, I was saying, ‘I’m very busy this week,’ and working it around, but that was fine.” (RR49)  “because of that flexibility that was built in, it didn’t actually disturb anything else.” (RR33)  “There’s an exercise course or an online exercise thing on Thursday morning. But I had returned to work by this point, and one of my shifts was directly in the middle of that, so I couldn’t. I couldn’t do that one just because, you know, timing was off, unfortunately.” (RR28)  “If they can continue to work and keep their social activities going or family appointments and things like that... I think it massively helps their recovery and improves their recovery.” (Staff S04)  “Even three sessions… they’re dedicating three days, usually, on iRehab over the week if they come to all three components.” (Staff01)  “Yeah, it’s so individualised. So, for example, one of the participants worked all during the day and found it really hard to get a private area to do his kind of weekly appointments and things, but was able to do an evening appointment. So we had some kind of evening appointments and things. It suited me fine, it suited them.” (Staff04) |
| **Perceived effectiveness** | **Tangible milestones as evidence -** Acceptability increased when participants could see or feel recovery happening. Everyday milestones, from lifting a kettle to walking further or climbing stairs, acted as tangible proof that rehabilitation was effective. | SC: Small gains like walking further or regaining independence marked recovery, but progress often felt slower or fragile.  RR: Structured measures (sit-to-stand tests, step counts, walking distances) provided visible evidence that reinforced the programme’s value.  Staff: Emphasised milestones as markers of acceptability, helping participants to recognise and believe in their progress. | “I’m driving again. I wasn’t allowed to drive for up to three months. I now drive again” (SC03)  “After about half a mile, I could faint. If there is something to sit on, I have to wait until the dizziness passes.” (SC06)  “I would like to get back to some normality, but I know I’m nowhere near going back properly yet. I’m just not, I’m not physically strong enough yet.” (SC19)  “My recovery has gone so quickly because I was on the treatment arm of iRehab” (RR11).  “I couldn’t walk for any distance… Now, I can walk more than three – I’ve just done a three-and-a-half-mile walk.” (RR02)  “I can sit and stand. When I first had my sit and stand test, I couldn’t do one, when I had my second one I did… seven or eight so that’s an amazing change.” (RR11)  “So they’ll be like getting back to work and things like that, they mightn’t be doing specifically what we’ve been doing but they’re more active and they’re doing more than what they were doing before the study. So it mightn’t be continuing on with exactly what we’re doing but it’s still going to be really beneficial in getting them back to where they were at.” (Staff02)  “They said their mobility is better. Since they started they can now stand up without using their arms, which they couldn’t do before. I think it’s just given more… They’ve said it’s given them more confidence about being able to do stuff and not necessarily worrying about it before they do it but trying it first.” (Staff03) |
|  | **Emotional and psychological gains -** Rehabilitation felt more effective when it supported emotional recovery alongside physical progress. Gains in resilience, hope, and renewed purpose strengthened participants’ belief in the value of rehabilitation. | RR: Described improved mood, greater self-compassion, acceptance of limits, and a renewed sense of purpose. Some highlighted how the programme helped reshape outlooks and support social re-engagement.  SC: Reflected on the emotional strain of ongoing symptoms, but also described moments of resilience, perspective change, or learning to live differently.  Staff: Observed improvements in emotional wellbeing and motivation, viewing these as central to participants’ sense that rehabilitation was valuable and acceptable. | “So I think it does need to be kind of participant-led and holistic, and look at a broader spectrum of things.” (Staff04)  “I get very depressed and I don’t understand why I get depressed, but I saw my doctor yesterday and he just said, “Because your mind hasn’t come to terms with this major shock you’ve gone through” (SC03)   “I don’t think about how I feel. [gets emotional] I just wish some of this stuff would stop.” (SC04)  I’m certainly, mentally, much better. I feel a lot better. (RR14)  “If you get a chance to do the programme, do it because it gives you your life back mentally, emotionally, and physically. It’s the best thing I’ve done.” (RR11).  “I was also encouraged to start building my social life back up and do things that would assist with how I was feeling mentally.” (RR12)  I almost see week one as like you’re presented with this caterpillar (Laughter) and then you’re giving it the nutrition and you’re giving it the care and attention it needs to build a cocoon around itself so they can float off like a butterfly (Staff01)  “In providing information to them that they might not already know, will hopefully help enhance or limit the barriers that they see in front of them to live that rich and meaningful life and get back to the physical, or get closer to the physical, and mental and functional capacity.” (Staff01)  “I’m fairly confident that for that population of people, their outcome measures will have improved a lot... from anecdotal experience, people have got a lot out of it and found a lot of benefit from it... physically and mentally.” (Staff07)  And I don’t think...if I hadn’t had the programme...I would have seen that for myself. I think I’d still be sitting around, feeling sorry for myself, thinking, Oh, I must do this, I must do that. (RR34) |
| **Self-efficacy** | **Knowing what to do and feeling safe to try** - Acceptability depended on participants feeling safe and equipped to engage with rehab activities without fear of harm. Participants described uncertainty about how far to push themselves, and valued guidance that helped them feel safe to start. | SC: Those with prior exercise habits could improvise and maintain activity, but many felt uncertain and unsure about how to progress without risking harm.  RR: Reported that iRehab provided the missing knowledge and structure, helping them move from fear to confidence and making participation feel worthwhile.  Staff: Emphasised the need for scaffolding and tailored advice to help patients feel confident, while also reflecting on their own learning and growing assurance in safely delivering iRehab. | “Earlier on, that would’ve helped, to talk to somebody, and to see what I was capable of doing. Because a lot of it is you’re scared to push yourself, in the beginning, to see actually how much you can do.” (SC01)  “...is it enough that I can lift my leg that far? Should they be helping me a bit, is it safe with the operation that you’ve had? And that’s, in my case, what I need, if you like. So I think that the conversation about your problem areas or concerns so that you’ve got an idea where to go with these things.” (SC04)  “If I’d been in a physiotherapy group sooner, they’d be guiding me, making sure I was doing what I needed to in order to get stronger” // That’s where I feel I need the support. I’m wary, like, what can I do without risking any damage.” SC08)  “It was the best bit. Or, like, knowing what to do, really. Yeah. And how to do it. And how to do it safely. Because that’s why I wasn’t feeling confident, before iRehab, to exercise, because I didn’t know what to do that wouldn’t hurt me, and that wouldn’t, you know, cause me any harm. I didn’t know how to exercise safely. And then I learned how to do it, and I became much better, much quicker.” (RR12)  I think I would’ve found it very difficult without the support and encouragement. To get started would’ve been really quite tricky because I was so weak and so feeble that I think I would’ve been almost frightened of it. Having the encouragement helped me to get going.” (RR14)  “Patients sometimes need that guidance and that… Not motivation but almost like, kind of, that, just, sharing of logical information as in like, “Well your legs might be…” I don’t know. “You might not have the balance, but we can do this seated, we don’t have to stand. You can take it a bit slower.” (Staff01)  “Every participant we’ve had has been very varied… we’re always learning on the job and having good discussions with each other and doing a bit of research to try to learn more about a particular condition or diagnosis.” (Staff02)  “If I’d been in a physiotherapy group sooner, they’d be guiding me, making sure I was doing what I needed to in order to get stronger (SC04) |
|  | **Belief in one’s own ability -** Rehabilitation was most acceptable when participants felt confident in their ability to progress. Visible improvements and goal achievement reinforced self-belief, while fragile confidence led to doubt and a need for reassurance. Staff also described their own confidence growing as they observed recovery. | SC: Some regained confidence by testing themselves (stairs, elastic bands), while others lost belief without support or feedback.  RR: Celebrated visible milestones, which boosted self-belief and commitment to the programme.  Staff: Gained confidence through seeing participant progress and reflecting on their own delivery | “And without them ringing on a regular basis, I wouldn’t have done anything, so I don’t think I’d be as far on as I am” (RR11)  “I set myself a goal to go to the shops which was a quarter of a mile and instead of walking half a mile I walked the whole mile. And I said I want to go to the cinema one night. Which I managed to do.” (RR05)  “So having a plan which was this rehab trial gave me some direction. So just, yes, it may not seem like a lot, but it made all the difference.” (RR21)  “I almost see week one as like you’re presented with this caterpillar (laughter) and then you’re giving it the nutrition and you’re giving it the care and attention it needs to build a cocoon around itself so they can float off like a butterfly, hopefully, by week six. It kind of does, for me, feel like that.” (Staff 01)  I think when you come out of ICU, patients lose trust in their own ability to make those decisions. And I think giving them that space to start gaining their own trust back...that they can maybe walk to the bottom of the garden and back, or they can, you know...that they can get that confidence in what they can do back themselves, I think is quite important. (Staff04)  I can remember waiting and waiting and waiting for somebody to come. And I just thought, I’m gonna just do this by myself. (SC35)  I’m still suffering from a lack of confidence in silly things. I get worried a lot when I never used to worry. (SC03)  That bit I find difficult, because I’m a little bit impatient hence now and then, I push my targets too far. I want to be on the golf course by the beginning of next year. At the moment, I know there are still a good few months to go, but I’m not 100% sure I’ll get there. (SC08)  I don’t think I’d have the confidence in my abilities like I do now. No, I’d be, I’d be sort of doubting myself. I would be doubting myself (RR29) |

## Additional file 10: iRehab trial investigators

**iRehab Trial Management Group (TMG)**

Brenda O’Neill PhD (Health Sciences, Ulster University), Danny McAuley MD (Critical Care Medicine, Queen’s University Belfast), Julie Bruce PhD (Clinical Trials, University of Warwick), Gavin Perkins DSc (Critical Care Medicine, University of Warwick), Martin Underwood MD (Clinical Trials, University of Warwick), Paul Dark MD (Critical Care Medicine, University of Manchester), Nigel Hart MD (Biomedical Sciences, Queen’s University Belfast), Ranjit Lall PhD (Statistics, University of Warwick), Chen Ji PhD (Statistics, University of Warwick), Mariam Ratna PhD (Statistics, University of Warwick), Mandana Zanganeh PhD (Health Economics, University of Warwick), Jason Madan PhD (Health Economics, University of Warwick), Katherine Jones PhD (Research Fellow, University of Warwick), Kerry Raynes MSc (Trial Manager, University of Warwick), Jill Costley BSc (Intervention Team, Ulster University), Annette Henderson BSc (Intervention Team, Ulster University), Darren Murphy MSc (Intervention Team, Ulster University), Rachel Clarke DClinPsy (Clinical Psychology, University Hospitals Plymouth NHS Trust), Cara Ghiglieri PhD (Process Evaluation Team, Queen’s University, Belfast), Bronwen Connolly PhD (Process Evaluation Team, Queen’s University Belfast), Judy Bradley PhD (Process Evaluation Team, Queen’s University Belfast), Roger Kenyon HNC (Patient and Public Involvement).

**iRehab Collaborators**

Below is a list of personnel who contributed to iRehab site activities. This list includes Principal Investigators, on behalf of their colleagues at sites.

Aintree University Hospital: Jane Snell (MBChB)

Altnagelvin Hospital: Sinead O’Kane (PGCert)

Birmingham Heartlands Hospital; Good Hope Hospital: Jonathan Weblin (MRes)

Craigavon Area Hospital: Denise McFarland (BSc)

Cumberland Infirmary: Jane Hedley (MSc)

Derriford Hospital: Kate Tantam (MRes)

Doncaster Royal Infirmary: Sarah Varga (PhD)

Freeman Hospital; Royal Victoria Infirmary: Catherine Donnison (MSc)

Glan Clwyd Hospital: Jennifer Davies (BSc)

Glangwili General Hospital: Samantha Coetzee (PGDip)

Great Western Hospital: Keeleigh Champman (MSc) & Amy Arnold (BSc)

Hereford County Hospital: Hannah Hayter (BSc)

James Cook University Hospital: Isabel Gonzales (FRCA)

James Paget University Hospital: Julie North (BSc)

King George Hospital; Queen’s Hospital: Mandeep Phull (FRCA)

Kingston Hospital: Eriko Morino (MBBCh)

Manchester Royal Infirmary: Deborah Paripoorani (MSc)

Musgrove Park Hospital: Patricia Doble (BSc)

Newham University Hospital; Whipps Cross Hospital: Juan Martin-Lazaro (PhD) & Charlotte Tai (FRCP)

North Devon District Hospital: Ruth Wood (BSc)

North Manchester General Hospital: Tracey Hodgkiss (RGN)

Northern General Hospital; Royal Hallamshire Hospital: Kathryn Birchall (RGN)

Pinderfields Hospital: Eva Hirst (PGCert)

Princess Royal Hospital: Denise Skinner (BSc)

Queen Alexandra Hospital: Kayode Adeniji (MSc)

Queen Elizabeth Hospital: Estefania Trues (MSc)

Rotherham Hospital: Cheryl Graham (BSc)

Royal Berkshire Hospital: Francesca Compton (BSc)

Royal Blackburn Hospital: Nicholas Truman (FFICM)

Royal Free Hospital: Lynn McDonnell (MSc) & Karthhika Jeyakumar (BSc)

Royal Glamorgan Hospital; Prince Charles Hospital: Bethan Gibson (MBBcH) & Daniel Law (BSc)

Royal Liverpool University Hospital: Alicia Waite (PhD)

Royal Oldham Hospital: Rebecca Chadwick (MSc)

Royal Sussex County Hospital: Daniel Hansen (BA)

Salford Royal Hospital: Elizabeth Howard (FICM) & Sarah Howe (BSc)

Southampton General Hospital: Zoe Van Willigen (MRes)

Stoke Mandeville Hospital: Philip Duggleby (FFICM)

Sunderland Royal Hospital: Anthony Rostron (PhD)

Ulster Hospital: Samantha Hagan (PGCert)

University Hospitals Coventry and Warwickshire: David McWilliams (PhD)

Warrington Hospital: Joanna Thomas (MRes)

Watford General Hospital: Ahmed Abdelaty (FFICM) & Valerie Page (FRCA)

West Cumberland Hospital: Kirstin Geer (MSc)

West Suffolk Hospital: Suzahn Wilson (MSc)

Whiston Hospital: Martin Udberg (MBChB)

Whittington Hospital: Anezka Pratley (DipHE)

Wrexham Maelor Hospital: Amy Charnock (MSc)

Wythenshawe Hospital: Luke Ward (BNurs)

Ysbyty Gwynedd: Ellen Knights (BSc)

## Additional file 11: Acknowledgements

**iRehab Sites**

There were 52 sites opened to recruitment for iRehab. These were:

Aintree University Hospital, Liverpool, UK

Altnagelvin Hospital, Londonderry, UK

Birmingham Heartlands Hospital, Birmingham, UK

Craigavon Area Hospital, Craigavon, UK

Cumberland Infirmary, Carlisle, UK

Derriford Hospital, Plymouth, UK

Doncaster Royal Infirmary; Bassetlaw Hospital, Doncaster, UK

Freeman Hospital; Royal Victoria Infirmary, Newcastle-Upon-Tyne, UK

Glan Clwyd Hospital, Wales, UK

Glangwili General Hospital, Wales, UK

Good Hope Hospital, Birmingham, UK

Great Western Hospital, Swindon, UK

Hereford County Hospital, Hereford, UK

James Cook University Hospital, Middlesborough, UK

James Paget University Hospital, Norfolk, UK

King George Hospital, London, UK

Kingston Hospital, Kingston-Upon-Thames, UK

Manchester Royal Infirmary, Manchester, England

Musgrove Park Hospital, Taunton, UK

Newham University Hospital, London, UK

North Devon District Hospital, Barnstaple, UK

North Manchester General Hospital, Manchester, England

Northern General Hospital/Royal Hallamshire Hospital, Sheffield, UK

Pinderfields Hospital, Wakefield, UK

Princess Royal Hospital, Haywards Heath, UK

Queen Alexandra Hospital, Portsmouth, UK

Queen Elizabeth Hospital, London, UK

Queen's Hospital, Romford, UK

Rotherham Hospital, Rotherham, UK

Royal Berkshire Hospital, Reading, UK

Royal Blackburn Hospital, Blackburn, UK

Royal Free Hospital, London, UK

Royal Glamorgan Hospital / Prince Charles Hospital, Wales, UK

Royal Liverpool University Hospital, Liverpool, UK

Royal Oldham Hospital, Greater Manchester, UK

Royal Sussex County Hospital, Brighton, UK

Salford Royal Hospital, Manchester, UK

Southampton General Hospital, Southampton, UK

Stoke Mandeville Hospital, Aylesbury, UK

Sunderland Royal Hospital, Sunderland, UK

Ulster Hospital, Belfast, UK

University Hospitals Coventry and Warwickshire, Coventry, UK

Warrington Hospital, Warrington, UK

Watford General Hospital, London, UK

West Cumberland Hospital, Whitehaven, UK

West Suffolk Hospital, Bury Saint Edmunds, UK

Whipps Cross Hospital, London, UK

Whiston Hospital, Prescot, UK

Whittington Hospital, London, UK

Wrexham Maelor Hospital, Wales, UK

Wythenshawe Hospital, Manchester, UK

Ysbyty Gwynedd Bangor, Wales, UK

iRehab was registered on the Associate PI Scheme, with six colleagues from sites completing this.

**We would like to thank the members of the:**

**Independent Data Monitoring and Ethics Committee (DMEC)**

Chair Dawn Teare, PhD (Biostatistics, Newcastle University), John Gormley, DPhil (Physiotherapy, Trinity College, Dublin), Catherine L Hough, MD (Pulmonary and Critical Care Medicine, Oregon Health and Science University).

**Trial Steering Committee (TSC)**

Chair John Saxton, PhD (Clinical Exercise Physiology, University of Hull), Ben Creagh-Brown, PhD (Critical Care, University of Surrey and Royal Surrey NHS Foundation Trust), Sue Jowett, PhD (Heath Economics, University of Birmingham), Ioana Marian, MSc (Statistics, University of Oxford), Paul Chazot, PhD (Patient and Public Involvement and Biosciences, Durham University).

**Patient Advisory Group (PAG)**

We would like to thank our Patient Advisory Group (PAG) members past and present for their inspiring contributions to this trial.

**Others supporting the iRehab trial**

Rowena Williams (TMG/Senior Project Manager, University of Warwick), Helen Bradley (TMG/Senior Project Manager,University of Warwick), Emma Padfield (TMG/Senior Project Manager, University of Warwick[OB1] [OB2] ), Radhika Kelkah (Document set-up/drafts, University of Warwick), Eva Schultz (telephone outcome assessment support, University of Warwick), Nicole De Valliere (administration, telephone outcome assessment support, University of Warwick), Jessica Smith (TMG/Senior Project Manager, University of Warwick), Susie Hennings (TMG/Senior Project Manager, University of Warwick), Caroline Wilson PhD (Process evaluation, Queen’s University Belfast), Kirsty Jerrard MSc (Intervention delivery, Ulster University), Niamh McCormac BSc (Intervention delivery, Ulster University), Kairenjit McCloy PhD (Intervention delivery, Ulster University), Michael Adams PhD (Intervention delivery, Ulster University), Alicia Clancy BSc (Intervention delivery, Ulster University),  *Gail Johnston (PPI training/support, Public Health Agency Northern Ireland), Louisa Edwards (TMG/Trial Coordinator,  University of Warwick), Raegan Barrows (University of Warwick), Sandra Baggott (TMG/Data Entry Clerk, University of Warwick), Sarah Lowe (TMG/Trial Manager, University of Warwick), Dalbir Kaur (TMG/Senior Project Manager, University of Warwick), Programming Team (Database development, reports, University of Warwick) and Quality Assurance Team (oversight, RAMP development, University of Warwick).
